# Neuromorphic Near‐Sensor and In‐Sensor Computing Enabled by Next‐Generation Material‐Based Sensors

**DOI:** 10.1002/advs.75742

**Published:** 2026-05-21

**Authors:** Su Yeon Jung, Gwang Ya Kim, Sejin Kim, Hyunjong Seo, Se Gi Lee, Sang Min Won, Dongjoon Rhee, Joohoon Kang

**Affiliations:** ^1^ Department of Chemical and Biomolecular Engineering Yonsei University Seoul Republic of Korea; ^2^ School of Advanced Materials Science and Engineering Sungkyunkwan University (SKKU) Suwon Republic of Korea; ^3^ School of Materials Science and Engineering Kookmin University Seoul Republic of Korea; ^4^ Department of Electrical and Computer Engineering Sungkyunkwan University (SKKU) Suwon Republic of Korea

**Keywords:** artificial sensory system, in‐sensor computing, multisensory perception, near‐sensor computing, neuromorphic sensor

## Abstract

The massive influx of continuous, real‐time environmental data demands highly energy‐efficient and low‐latency sensory processing. Conventional artificial sensory systems are limited by severe data transfer overhead issues due to physically separated processing and memory units, coupled with analog‐to‐digital converters. To resolve these issues, neuromorphic sensory platforms inspired by the biological nervous system have emerged as an innovative paradigm. This Review comprehensively investigates the structural evolution and current research trends of neuromorphic near‐sensor and in‐sensor computing systems. Initially, the fundamental physical mechanisms underlying artificial neurons and synapses are systematically analyzed. Furthermore, the distinct operating principles of optical, mechanical, and chemical sensors corresponding to the five human senses are discussed. To establish a clear structural framework, we systematically categorize neuromorphic‐integrated sensory systems into near‐sensor and in‐sensor computing architectures based on their level of integration. Near‐sensor processing minimizes data movement through system‐level integration, whereas in‐sensor computing executes stimulus transduction and state evolution simultaneously at the device level. Based on this classification, we extensively discuss recent research trends of near‐sensor and in‐sensor computing tailored to each of the five human senses. Ultimately, by identifying domain‐specific bottlenecks, this article provides strategic material and architectural guidelines for realizing fully integrated, next‐generation artificial cognitive systems.

## Graphical abstract

This Review presents a structural framework that classifies neuromorphic sensing into near‐sensor and in‐sensor architectures, clarifying physical coupling between sensing and computation. The framework connects neural and synaptic device functions with recent advances in optical, mechanical, and chemical sensing, compares energy consumption and response speed, and outlines key limitations and future research directions.

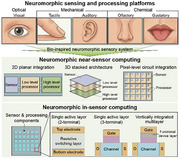

## 1 Introduction

**FIGURE 1:**
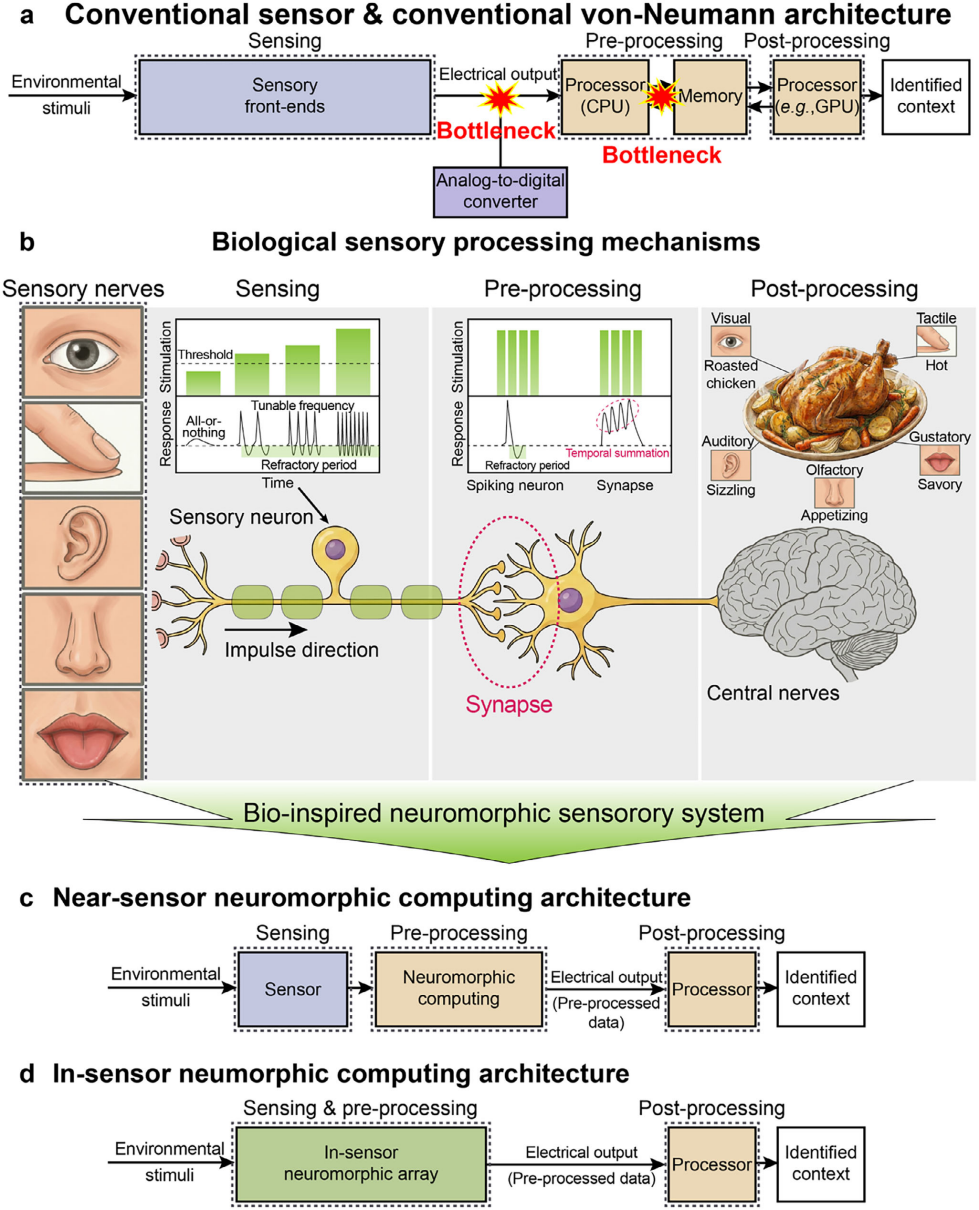
Bio‐inspired neuromorphic sensor architectures. (a) The stimuli processing flow in a conventional sensor system based on the von Neumann architecture. (b) Biological sensory processing mechanisms for the human senses. This panel illustrates how sensory neurons encode environmental stimuli into efficient spiking signals, characterized by threshold‐driven firing, all‐or‐nothing responses, spiking frequency modulation, and refractory periods. The spike signals undergo pre‐processing via temporal summation at the synapse before being transmitted to the central nervous system (brain) for complex perception, such as identifying a roasted chicken through multimodal sensory integration. The stimulus processing flow of biomimetic artificial receptors: (c) near‐sensor neuromorphic system and (d) in‐sensor neuromorphic system.

The growing demand for automated systems across diverse industrial sectors, driven by the rapid development of artificial intelligence (AI) and the internet of things (IoT), underscores the importance of accurately perceiving environmental stimuli (e.g., light, pressure, and chemical signals) and rapidly processing input data to enable context‐aware adaptive responses [1, 2, 3]. The core component enabling this function is the artificial sensory system, which is composed of sensors that detect and transduce environmental inputs into electrical outputs and computational networks that interpret and process the resulting data. As the automated systems continue to mature, a paradigm shift from passive data‐collecting nodes to intelligent sensing architectures capable of both capturing and actively processing large volumes of information in real time becomes imperative. However, conventional sensory systems are fundamentally limited in this regard due to their underlying architectural constraints. In particular, analog signals from front‐end sensors must be converted into digital signals by a separate analog‐to‐digital converter (ADC) circuit before they can be processed by the computing unit (Figure 1a) [4]. The inclusion of an ADC inevitably introduces additional data transfer between the sensor and the computing unit, resulting in increased latency and limited data processing speed [5, 6, 7]. Furthermore, because the ADC digitizes not only meaningful information but also noise and redundant signals, they generate vast amounts of raw data, degrading signal fidelity and reducing overall processing efficiency [8]. Another limitation originates from the separation of memory and processing units, which necessitates frequent data transfers in which digital signals are repeatedly stored, processed, and rewritten to memory, resulting in the so‐called von Neumann bottleneck [4]. The massive movement of data through the transmission bus causes unnecessary time delay, high energy consumption, and heat generation that can lead to chip degradation [9, 10, 11]. Moreover, because the processing speed of processors significantly exceeds the data transfer rate of memory, processors are forced to wait for data, resulting in increased latency and unnecessary power consumption [12, 13]. This issue is further exacerbated as continued advancements in semiconductor technology widen performance gap between the processor and memory [14].

To overcome these limitations, neuromorphic technology has emerged as a promising alternative, mimicking the structure and signal processing mechanisms of the human brain in hardware [15, 16]. The human nervous system consists of neurons and synapses that enable efficient information transmission and processing. In particular, neurons integrate signals received from the surrounding synapses and transmit signals to the next neuron only when the accumulated electric potential exceeds a specific threshold [17]. Specifically, electric potentials received through dendrites accumulate across the neuronal membrane, and once this potential exceeds a threshold, a transient pulse known as an action potential is generated and subsequently transferred to adjacent neurons. Synapses play a dual role by not only adjusting the strength and duration of neuronal signals but also modulating connection strength through synaptic plasticity, which serves as the fundamental mechanism for learning and memory [18]. Upon receiving inputs, synapses regulate signal transmission to downstream neurons by enhancing important signals and suppressing noise. They also support learning and memory through activity‐dependent plasticity, strengthening frequently activated connections while weakening those with low activation frequency.

By implementing biological neurons and synapses in hardware (Figure 1b), neuromorphic sensing and computing provide an effective solution to the limitations of conventional sensory systems relying on the von Neumann architecture. Unlike conventional systems, where the processor and memory are separated, neuromorphic computing architectures integrate computing and storage within the single platform, thereby alleviating the von Neumann bottleneck [19]. Furthermore, while conventional systems process data sequentially, neuromorphic counterparts enable massively parallel processing, thereby reducing latency substantially [20]. In the context of sensing, neuromorphic sensors operate on an event‐driven basis, generating output signals only in response to meaningful changes in the environment [21]. This strategy significantly lowers power consumption, reduces noise, and allows for in‐sensor pre‐processing, which reduces the computational burden on the processor and facilitates faster data transmission [22].

To further overcome the limitations of the conventional sensory systems, emerging architectural strategies—namely neuromorphic near‐sensor and in‐sensor computing—have gained increasing attention (Figures 1c,d). Near‐sensor computing features physically distinct sensing and computing elements that are placed in extremely close proximity, often connected through vertical interconnects rather than long‐distance buses, enabling immediate calculation upon signal generation (Figure 1c) [23, 24, 25]. In contrast, in‐sensor computing integrates sensing and processing within a single device, such that the sensing device itself performs computations directly after stimulus detection without relying on a separate processor [8, 26, 27]. These architectures enhance data accuracy by removing unnecessary noise through pre‐processing at the sensor unit and achieve superior energy efficiency and minimal latency by eliminating or minimizing the physical distance between the sensing and computing units [28]. Nevertheless, the distinction between near‐sensor and in‐sensor computing remains insufficiently defined in the current literature. In prior studies, the two terms have been used interchangeably or introduced without consistent architectural criteria, particularly with respect to device configuration, array‐level integration, and circuit functionality [29]. Such inconsistency makes the comparison of reported systems difficult and obscures the design trade‐offs associated with each computing paradigm. Therefore, this article proposes a clear classification framework for near‐sensor and in‐sensor computing, with the aim of establishing consistent terminology and providing a practical guide for the analysis and design of AI‐enabled sensor systems.

Herein, we review the operating principles, structures, and current research directions of neuromorphic in‐sensor and near‐sensor computing architectures enabled by next‐generation materials for mimicking the five human senses. We begin by introducing the fundamental principles of the two core components of a neuromorphic sensory platform: (1) neuromorphic computing systems and (2) sensors. In the section on neuromorphic computing, we examine how artificial devices mimic the operations of biological neurons and synapses, as well as the underlying mechanisms that enable such behavior (Section 2). We then describe how artificial sensors reproduce the five primary human sensory modalities by categorizing them into optical sensing for vision, mechanical sensing for touch and hearing, and chemical sensing for olfaction and gustation (Section 3). Next, we discuss neuromorphic near‐sensor and in‐sensor computing systems, focusing on their operating principles and differences in architecture and integration scale (Section 4). Finally, we review recent advances in neuromorphic in‐sensor and near‐sensor computing applications realized through next‐generation sensing materials, which are organized according to the five primary human senses (Section 5), and conclude by outlining future development directions and evaluating their technological potential (Section 6).

## 2 Background of Neuromorphic Computing Systems

### 2.1 Fundamental Elements of Neuromorphic Computing Systems

#### 2.1.1 Artificial Neurons: Signal Encoding Principles and Characteristics

In neuromorphic computing systems for sensor applications, the artificial neuron serves as the primary signal encoder, which is functionally equivalent to biological sensory receptors or afferent nerve fibers. Its core function is analog‐to‐spike conversion where continuous analog signals generated by sensors are transformed into discrete spike trains [30]. This process effectively replaces the high power‐consuming ADCs in conventional systems and enables event‐driven processing. To emulate these dynamic neural behaviors, memristive devices—memory devices with either two or three terminals whose resistance depends on the history of applied electrical stimuli—are employed. In particular, volatile memristive devices exhibiting fast switching speeds, threshold switching characteristics, and volatility are highly advantageous. Their ability to rapidly transition to a low‐resistance state (LRS) and spontaneously recover to an initial high‐resistance state (HRS) provides a physical platform for implementing artificial neurons by emulating the automatic resetting mechanism of biological neurons after a spike event. To effectively translate sensory information, artificial neurons must exhibit several key biological characteristics. First, they operate according to the all‐or‐nothing law where neuron fires a spike only when the accumulated input exceeds a specific threshold [31]. Furthermore, the amplitude of the generated spikes is constant regardless of the stimulus intensity. Instead, the magnitude of the external stimulus is encoded in the frequency of the spikes, a mechanism known as rate coding [32]. For instance, stronger optical or pressure input results in a higher firing frequency, which allows the system to process magnitude information with binary spikes. This dynamic behavior is typically described by the leaky integrate‐and‐fire (LIF) model. The neuron does not respond to inputs instantaneously but accumulates the incoming charge over time, mimicking the temporal summation of sensory signals and effectively filtering out high‐frequency noise [33]. A spike is fired only when the membrane potential reaches a critical threshold. Furthermore, unlike permanent storage, the membrane potential naturally decays over time if no input is added. This leaky characteristic, derived from the volatile nature of the device, is vital for real‐time sensing because this feature ensures that the system does not retain outdated sensory information and returns to a resting state in the absence of stimuli. Finally, immediately after generating a spike, the neuron enters a refractory period characterized by temporary unresponsiveness to further stimuli while resetting to its initial state [34]. In sensory applications, this period plays two critical roles: (1) limiting the maximum firing rate to prevent system saturation under extreme stimuli and (2) ensuring the unidirectional signal propagation between the sensor and the processing unit, thereby preventing backward flow.

#### 2.1.2 Artificial Synapses: In‐Memory Processing Principles and Characteristics

While artificial neurons function as signal encoders, artificial synapses serve as the core units for memory and synaptic weight updates, playing a role analogous to the neural networks in the biological brain. Different behaviors of neurons are emulated by using distinct types of memristive devices. For example, long‐term potentiation (LTP) and long‐term depression (LTD) are typically mimicked by non‐volatile memristive devices exhibiting tunable conductance and stable memory retention, whereas short‐term plasticity (STP) is realized using volatile memristive devices. These characteristics enable in‐memory computing, where data storage and processing occur simultaneously. The colocalization of data storage and processing within a single architecture enables the system not only to store sensory information but also to evaluate the importance of signals, recognize patterns, and perform pre‐processing directly at the sensor node. To implement these functions, the device must exhibit analog conductance modulation capabilities. Unlike binary switches that represent only “0” and “1,” artificial synapses must provide multi‐level states to simulate synaptic weights. A higher number of accessible conductance states allows for superior computational precision and reduced quantization errors during the processing of complex sensory data.

The fundamental mechanisms for learning are LTP and LTD, which involve non‐volatile conductance modulation that persists over time [35]. These characteristics enable the long‐term storage of learned information. For effective learning, the linearity and symmetry of the weight update are critical. Low linearity can lead to non‐uniform learning, where early training data are overweighted while later data are neglected. Furthermore, asymmetric update characteristics where the potentiation and depression rates differ can cause the device to reach saturation too quickly or make conductance tuning difficult, thereby degrading the classification accuracy of the sensor system. In addition to long‐term behavior of artificial synapses, STP is essential for dynamic signal processing. The device emulates STP by a temporary change in conductance level that spontaneously returns to its initial state over time [36]. A representative behavior is paired‐pulse facilitation (PPF), where the postsynaptic response increases when presynaptic spikes arrive in succession. In sensory applications, PPF acts as a hardware‐level noise filter by amplifying repetitive and significant environmental signals while ignoring sporadic and random noise. Typically, synaptic devices exhibit a transition from STP to LTP depending on the pulse intensity and repetition rate, allowing the system to distinguish between temporary fluctuations (noise) and persistent information (memory) [37, 38, 39]. Finally, to learn the temporal relationships of sensory inputs, the device must emulate spike‐timing‐dependent plasticity (STDP) [40]. This rule adjusts the synaptic weight based on the relative timing of the pre‐synaptic and post‐synaptic spikes. If a pre‐synaptic spike precedes a post‐synaptic spike, the connection is strengthened (potentiation), implying a causal relationship [41]. Conversely, if the order is reversed, the connection is weakened (depression). This characteristic is particularly essential for motion detection or sequence learning in vision and auditory sensors, where the timing of events carries significant information.

### 2.2 Physical Mechanisms of Neuromorphic Devices

#### 2.2.1 Filamentary Switching (ECM, VCM)

Filamentary switching represents the most widely investigated mechanism in two‐terminal memristive devices based on metal‐insulator‐metal (MIM) structure, characterized by the formation and rupture of conductive filaments within an insulating resistive switching layer [42]. The MIM devices exhibiting this resistive switching mechanism are commonly referred to as resistive random‐access memory (RRAM) devices or memristors. The fundamental principle of conductive filament formation and rupture involves the migration of metal cations or oxygen vacancies through defect sites, such as grain boundaries, under an applied electric field [43]. The simple MIM structure of RRAMs allows for high‐density integration in crossbar arrays, making such devices an ideal candidate for scalable neuromorphic sensory architectures where high spatial resolution is required, such as in artificial skins or retina‐like image sensors [44, 45]. Another unique advantage of these devices in neuromorphic applications is their reconfigurability between neuronal and synaptic functions. Depending on the strength of the conduction filament, the device can exhibit either volatile or non‐volatile switching characteristics [46, 47]. Weak filaments tend to self‐rupture spontaneously when the external bias is removed, mimicking the “integrate‐and‐fire” and “leaky” dynamics of biological neurons. Conversely, strong filaments maintain their connection without bias, enabling the storage of synaptic weights. Recent studies have demonstrated that these two distinct modes can coexist within a single device by controlling the current compliance (*I*_CC_) during the electroforming process [48]. A low *I*_CC_ allows for the formation of unstable, volatile filaments suitable for signal encoding, while a high *I*_CC_ induces robust, non‐volatile filaments for memory storage [49, 50]. This tunability allows a single sensory hardware platform to dynamically adapt its function between sensing and processing based on operational needs.

**FIGURE 2:**
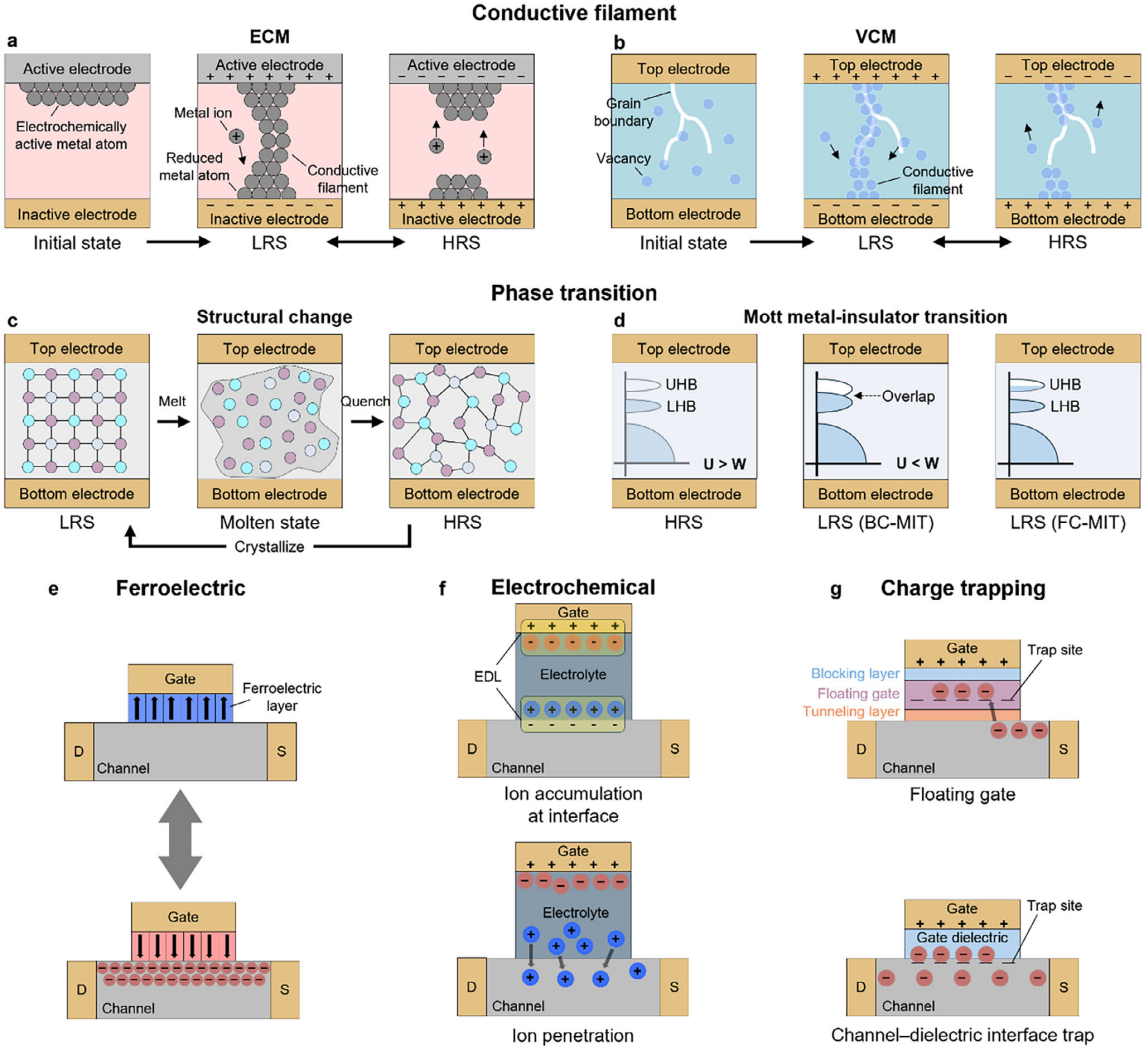
Working mechanisms of the neuromorphic device. Resistive switching via conductive filament formation in (a) electrochemical metallization (ECM) and (b) valence change mechanism (VCM), driven by oxygen vacancy migration along grain boundaries. (c) Phase transition between amorphous and crystalline states. (d) Mott metal‐insulator transition (BC‐MIT and FC‐MIT). (e) Threshold voltage modulation via ferroelectric polarization in a FeFET. (f) Conductance modulation mechanisms in electrolyte‐gated FETs classified by ion permeability: ion accumulation (electrostatic gating) and ion penetration (electrochemical doping). (g) Conductance modulation mechanisms mediated by charge trapping/de‐trapping at trap sites.

One mechanism underlying filamentary switching is electrochemical metallization (ECM) (Figure 2a). RRAM devices based on the ECM effect, also referred to as conductive‐bridge random‐access memory (CBRAM), typically employ an electrochemically active anode (such as Ag, Cu, or Ni) and an inert cathode [51]. When a positive voltage is applied, metal atoms at the active electrode are oxidized into cations (Ag^+^, Cu^2+^), which migrate through the solid electrolyte and are reduced at the cathode to form a metallic filament. ECM is characterized by a high on/off ratio due to the metallic nature of the bridge. In sensory systems, this distinct contrast is advantageous for implementing threshold‐based switches or high‐sensitivity neurons that must clearly distinguish between a signal event and background noise. Common electrolyte materials that facilitate ion migration while remaining electrically insulating include metal oxides (SiO_2_, HfO_2_) and amorphous chalcogenides (GeS, GeS_2_, Ag_2_S), which are particularly suited for integration with photonic and ionic sensors [46, 52, 53].

As another filamentary switching mechanism distinct from ECM, the valence change mechanism (VCM) relies on the migration of intrinsic oxygen vacancies (*V*_O_) or anion defects within the switching oxide and employs inert metals for both electrodes (Figure 2b). The conductive filament is formed by the accumulation and rearrangement of these vacancies, often modulating the Schottky barrier height at the electrode interface [54]. Because the filament consists of intrinsic defects within the crystal lattice, VCM devices generally exhibit superior stability and retention compared to ECMs. This stability allows for precise analog conductance modulation, making VCM particularly beneficial for implementing artificial synapses that require high linearity and symmetry for accurate in‐sensor computing [55]. Typical resistive switching materials include transition metal oxides (WO*_x_*, TiO_2_) and various two‐dimensional (2D) materials (MoS_2_, WS_2_, h‐BN) [56, 57, 58, 59, 60, 61]. Furthermore, since oxygen vacancy dynamics are sensitive to the ambient atmosphere, VCM devices can also be engineered to function as intrinsic gas or moisture sensors by exposing the switching layer to the environment.

#### 2.2.2 Phase Transition (Structural Change, MIT)

Beyond filamentary switching, phase transition mechanisms utilize the physical state change of the material itself to store information or generate spikes. These mechanisms are broadly categorized into structural phase changes involving atomic rearrangement and metal‐insulator‐transition (MIT) involving the modification of the energy band structure.

Structural phase change memory exploits the significant resistance contrast between the amorphous and crystalline phases (Figure 2c) [62]. The amorphous phase exhibits high resistance while the crystalline phase shows low resistance [63, 64, 65, 66]. The switching process is thermally driven by Joule heating induced by external voltage pulses [67]. To reset the device to the amorphous state, a short and high‐amplitude pulse is applied to melt the resistive switching material slightly above its melting point [68]. This is followed by rapid quenching to room temperature, which prevents the atoms from reorganizing and effectively freezes them in an amorphous structure (HRS). Conversely, setting the device to the crystalline state involves a longer and moderate amplitude pulse to anneal the switching layer at a temperature between the crystallization and melting points. During this process, atoms have sufficient time to rearrange into an ordered crystalline structure (LRS) [68]. A key advantage of structural phase change memory is its capability for multi‐level storage. The ratio of crystalline to amorphous volumes is modulated by precisely controlling the input energy [68]. This tunability enables the high‐precision synaptic weights required for neuromorphic computing. Despite high switching speed and stability, this mechanism faces challenges such as high‐power consumption due to the significant heat required for the melt‐quench process. Another issue is resistance drift where the resistance of the amorphous state gradually increases over time due to structural relaxation [69]. However, the unique material properties offer distinct advantages for sensory applications. Specifically, GST(Ge_2_Sb_2_Te_5_) exhibits high responsivity to light, making this phase‐change material an excellent component for optical sensing [70]. Therefore, GST is being actively investigated for all‐photonic synapses capable of directly processing signals from optical sensors without electrical conversion. Emerging 2D transition metal dichalcogenides (TMDCs), such as MoTe_2_, exhibit a reversible phase transition between a semiconducting 2H‐phase and a metallic 1T‐phase under applied electric fields and are gaining attention due to their mechanical flexibility [71]. These 2D materials offer a pathway for implementing ultra‐thin and flexible neuromorphic devices suitable for artificial skin applications.

Distinct from structural atomic rearrangement, MIT is an electronic phase transition prominently observed in a specific class of materials known as Mott‐insulators (Figure 2d). In these materials, repulsion between electrons dominates over their kinetic energy. This strong interaction forces electrons to localize at atomic sites rather than moving freely. Consequently, the energy band splits into a lower Hubbard band (LHB) and an upper Hubbard band (UHB) [72]. This splitting creates an energy bandgap that forces the material to behave as an insulator despite having partially filled bands. When an external stimulus creates enough energy to overcome the repulsive force between electrons, the material undergoes a sharp transition to a metallic state [73]. Depending on the specific driving mechanism, this transition manifests as either volatile or non‐volatile switching. One mechanism responsible for the MIT is the bandwidth‐controlled MIT (BC‐MIT). This mechanism is typically triggered by external physical stimuli, such as voltage and strain, which compress the interatomic distance. This compression generates an orbital overlap and increases the electron bandwidth. As a result, the kinetic energy increases until the Hubbard gap collapses [74]. Crucially, this transition is volatile: once the stimulus is removed, the material spontaneously cools and recovers its insulating state. This behavior perfectly mimics the “integrate‐and‐fire” and “leak” dynamics of biological neurons. Thus, Mott insulators like VO_2_ and NbO*_x_* are ideal candidates for neuron devices [75, 76, 77, 78]. Another mechanism underlying the MIT is the filling‐controlled MIT (FC‐MIT). This transition is achieved by doping the material with ions. The injected charge carriers shield the strong electron‐electron repulsion, which causes the gap between the UHB and LHB to collapse. Since the dopant ions remain in the lattice even after the external bias is removed, the low‐resistance metallic state is maintained [73]. This non‐volatile characteristic allows Mott materials to function as synaptic memory. Furthermore, the MIT mechanism offers a unified material platform that can support both neuronal and synaptic functions within a single sensor array. Representative materials for this mechanism include Pr*_x_* Ca_1−_ *_x_* MnO_3_ (PCMO) and SmNiO_3_ [79, 80].

#### 2.2.3 Ferroelectric (FTJ, FeFET)

Ferroelectric switching utilizes the spontaneous electric polarization of ferroelectric materials to store information or generate signals. Unlike common insulators, ferroelectrics possess a remnant electric dipole moment even in the absence of an external stimulus, enabling information storage in the direction and magnitude of the remnant polarization. The microscopic mechanism of this polarization varies depending on the atomic structure of the ferroelectric material. In the traditional perovskite oxides (ABO_3_), the B‐site cation located at the center of the unit cell shifts from the central axis to one of two stable positions to form a polarization [81]. In contrast, in hafnium oxide‐based ferroelectrics, polarization is induced by the specific displacement of four oxygen anions within the unit cell [82]. When an external voltage is applied, the direction of these dipoles can be flipped up or down. Even after the voltage is removed, the direction is maintained, which results in a hysteresis loop and ensures non‐volatile characteristics. Furthermore, the conductivity can be finely tuned through partial domain switching. This capability allows for analog switching, which is essential for neuromorphic computing [83].

A common type of memory device that uses ferroelectrics is the ferroelectric tunnel junction (FTJ), which is a two‐terminal device with a ferroelectric insulator that serves as the tunneling barrier [84]. The FTJ operates based on the tunnel electro‐resistance (TER) effect, in which the polarization direction of the ferroelectric layer modulates both the width and height of the ferroelectric/electrode interfacial energy barrier that electrons must pass through. When the energy barrier weakens due to polarization alignment, electrons can tunnel through the electrode, which allows current to flow [33, 83]. To induce this tunneling effectively, the device is implemented with an ultra‐thin ferroelectric film typically less than 5 nm thick [85]. FTJs offer significant advantages including picosecond (ps)‐level ultrafast switching and low energy consumption ranging from femtojoules to picojoules (fJ–pJ) [85, 86]. Additionally, they possess intrinsic non‐linear *I*–*V* characteristics which reduce leakage current. However, they have disadvantages such as a relatively low on‐current, which leads to a low on/off ratio and a slow read‐out speed [87].

The ferroelectric field‐effect transistor (FeFET) is another type of widely used ferroelectric memory device. This device features a three‐terminal architecture and typically adopts a gate‐ferroelectric‐dielectric‐semiconductor (MFIS) structure (Figure 2e). The operation principle involves applying a write voltage to the gate to induce polarization within the ferroelectric layer. This polarization causes electrons to accumulate in the channel, which results in a low threshold voltage (*V*_th_) and current flow (LRS). Upon removal of the applied voltage, the ferroelectric polarization is retained, allowing the device to remain in the LRS. Subsequently, if an erase pulse is applied, the ferroelectric is polarized in the opposite direction. This process causes the electrons in the channel to deplete and transitions the device to a high threshold voltage or HRS [88, 89]. FeFET is characterized by nanosecond (ns) level switching speeds and low power consumption [81]. Based on these non‐volatile characteristics, they are highly suitable for implementing synaptic devices. A notable drawback is that defects at the ferroelectric/insulator interface can cause device degradation, which leads to relatively lower endurance [90].

Regarding material selection, early research primarily focused on perovskite oxide materials, such as PbZr*_x_* Ti_1−_ *_x_* O_3_ (PZT) and BaTiO_3_ (BTO) [81, 91]. However, recent developments have shifted toward fluorite structures, specifically HfO_2_‐based materials including Hf_0.5_Zr_0.5_O_2_ (HZO), Si‐doped HfO_2_, and Al‐doped HfO_2_ [81, 92, 93, 94, 95, 96]. These materials are particularly advantageous because they are fully compatible with standard CMOS processes. Additionally, organic materials such as poly(vinylidene fluoride‐trifluoroethylene) (P(VDF‐TrFE)) are gaining attention [86]. Since these materials act as both ferroelectric and excellent piezoelectric, mechanical pressure can alter the dipole moments within the crystal structure to induce polarization. This unique property allows them to be applied as intrinsic pressure sensors capable of detecting tactile information [97].

#### 2.2.4 Electrochemical FET

Electrochemical field‐effect transistors (EC‐FETs) typically adopt a three‐terminal structure consisting of a source, a drain, and a channel, with an electrolyte positioned between the channel and the gate. A defining characteristic of this electrolyte is its ionically conducting nature while remaining electronically insulating [98]. Unlike conventional dielectric layers, the mobile ions within the electrolyte play a crucial role in modulating the channel conductance. The operating mechanism is primarily classified into two distinct modes based on whether the ions within the electrolyte accumulate at the interface (electrostatic gating) or penetrate the channel material (electrochemical doping) (Figure 2f) [99, 100].

Electrostatic gating in EC‐FETs arises from ion accumulation at the electrolyte/channel interface without ion penetration into the channel. When a voltage is applied, ions in the electrolyte migrate and accumulate at the electrolyte/gate and electrolyte/channel interfaces. The accumulated ions form an electric double layer (EDL) with high areal capacitance, typically exceeding that of conventional FETs based on solid‐state gate insulators. The resulting strong electric field induces the accumulation of charge carriers (electrons or holes) in the channel to balance the ionic charge, thereby modulating the conductance [101, 102]. When the external electric field is removed, the accumulated ions diffuse back to their original equilibrium state. Consequently, the channel current recovers to its initial level, exhibiting volatile characteristics, which can be used to implement STP [103, 104, 105]. Furthermore, the relaxation dynamics of the ions mimic the “leaky” behavior of biological membranes making this mechanism suitable for LIF neuron devices [106, 107].

In contrast, electrochemical doping results from the ion penetration into the channel of EC‐FETs, which typically occurs when a relatively strong voltage is applied to the gate. In this process, cations from the electrolyte are driven into the interior or bulk of the channel material. This physical insertion alters the oxidation state or doping level of the channel and directly modulates its conductivity [108]. This process is fundamentally different from charge accumulation as the ions penetrate the surface. Crucially, the penetrated ions do not spontaneously diffuse out of the channel even after the electric field is removed [109]. Therefore, the device exhibits non‐volatile characteristics where the modulated conductivity is retained. This property makes electrochemical doping an ideal mechanism for implementing LTP in synaptic devices, effectively mimicking the permanent weight changes in biological synapses [110].

A significant advantage of EC‐FETs is the ability to induce both volatile and non‐volatile mechanisms within a single device by controlling the magnitude or duration of the applied voltage. Beyond computing, EC‐FETs possess high compatibility with biological environments, such as aqueous solutions [111]. This feature makes them highly appropriate for application as chemical or biological sensors capable of directly interfacing with living tissues. Representative electrolyte materials include lithium‐based solid electrolytes such as LiPON, organic conductive polymers like poly(3,4‐ethylenedioxythiophene):poly(styrenesulfonate) (PEDOT:PSS), and ceramics such as yttria‐stabilized zirconia (YSZ) [110, 112, 113]. These materials are selected based on their ionic conductivity and compatibility with the channel material to optimize the switching speed and stability of the device.

#### 2.2.5 Charge Trapping/Detrapping

Charge trapping and de‐trapping mechanisms modulate the conductivity of a device by capturing or releasing charge carriers at specific defect sites within the insulating layer or at the interfaces. Unlike filamentary switching which relies on the formation of a physical conductive path, this mechanism depends on the electronic modification of the energy barrier. Consequently, these devices typically exhibit electroforming‐free behavior and self‐rectifying characteristics in two‐terminal memristors [114, 115, 116]. These features effectively suppress sneak path currents in crossbar arrays and allow for analog conductance modulation with low operation voltages. To realize this barrier modification at the device level, charge‐trapping concepts are widely implemented in both two‐terminal and three terminal configurations.

In two‐terminal memristor structures, the device generally consists of a simple MIM configuration. The switching mechanism is primarily driven by the modulation of the Schottky barrier at the electrode/switching layer interface. When metal oxide is used as the switching layer, charge carriers injected from the electrode are trapped in deep‐level defects such as oxygen vacancies within the oxide or near the interface [114, 117]. These trapped charges create a localized internal electric field, which alters the height and width of the Schottky barrier or the tunneling resistance, effectively controlling the current flow across the junction. In the case where a two‐dimensional (2D) material is used as a single switching layer, the operating principle relies heavily on interface states due to the atomically thin nature of the material. Structural defects such as sulfur vacancies or grain boundaries act as effective charge trap sites. When charge carriers are trapped at these interface defects, they modulate the Schottky barrier height (SBH) and width at the contact, thereby controlling the carrier injection efficiency without the need for an additional oxide layer [118].

In three‐terminal transistor configurations, the mechanism is categorized based on the location of the charge traps (Figure 2 g). The first type resembles floating gate flash memory, which consists of a gate/blocking layer/floating gate/tunneling layer/channel structure. In this configuration, charges tunnel through the thin tunneling layer and are stored in the isolated floating gate, creating an electric field that shifts the threshold voltage (*V*_th_) [119, 120, 121]. Crucially, recent studies actively exploit large charge storage capacity of the floating gate to finely tune the channel conductance based on the amount of trapped charge, enabling multiple analog states [122]. The second type utilizes interface‐trapping in standard MOSFET structures. Here, the mechanism is based on charge traps at the dielectric‐channel interface. These traps modulate *V*_th_ to induce hysteresis and thereby control the channel conductivity [123, 124]. Both architectures can function as synaptic devices by precisely controlling the amount of trapped charges to emulate the gradual weight updates of biological synapses.

In summary, the selection of an appropriate neuromorphic device for specific sensory applications requires a comprehensive understanding of the inherent trade‐offs among various physical mechanisms. As summarized in Table 1, each mechanism exhibits distinct characteristics—including specific advantages and limitations in key metrics such as switching speed, endurance, retention, and operating voltage—which subsequently demonstrates its suitability for particular sensing modalities. For instance, mechanisms like phase transition memory are highly suitable for optical sensing architectures, while filamentary switching and ferroelectric devices are frequently employed in both optical and mechanical sensory systems where rapid signal processing is crucial. Conversely, electrochemical devices, despite having relatively slower switching speeds, can operate at low voltages and utilize ion migration dynamics, making them advantageous for chemical sensing and bio‐interfacing applications. Ultimately, carefully matching these fundamental physical mechanisms with the specific requirements of the target application—whether optical, mechanical, or chemical—is an essential prerequisite for optimizing the performance, latency, and energy efficiency of next‐generation near‐sensor and in‐sensor computing platforms.

**TABLE 1:** Comparing key aspects of physical mechanisms for neuromorphic characteristics [125, 126, 127, 128, 129, 130, 131, 132, 133, 134, 135, 136, 137, 138, 139, 140, 141, 142, 143, 144, 145, 146, 147, 148, 149, 150].

Mechanism	Advantages	Disadvantages	Sensing applications
Filamentary switching (ECM, VCM)	High switching speed (10–100 ns) Low operating voltage (1–3 V) Low energy consumption (1–10 pJ)	High device‐to‐device variability Low endurance (10^3^–10^4^ cycles) Low linearity	Optical [125, 126] Mechanical [127, 128, 129] Chemical [130, 131]
Phase transition (structural change, MIT)	High retention (∼10 years) High switching speed (10–100 ns)	Low linearity High reset temperature (> 400°C) Resistance drift	Optical [132, 133, 134]
Ferroelectric	Low energy consumption (10 fJ–1 pJ) High switching speed (10–100 ns) Multilevel conductance states	High operating voltage (5–10 V) Scaling limitation Endurance‐retention trade‐off	Optical [135, 136, 137] Mechanical [138, 139, 140]
Electrochemical	High linearity and symmetry Low operating voltage (∼1 V) Multilevel conductance states	Low switching speed (100 µs–100 ms) Low compatibility with CMOS	Mechanical [141, 142, 143] Chemical [144, 145, 146]
Charge trapping	High compatibility with CMOS Multilevel conductance states	High operating voltage (∼10 V) Low endurance (10^3^–10^4^ cycles)	Optical [147, 148] Mechanical [149, 150]

## 3 Sensing Mechanisms of Artificial Sensory Systems

A sensor is defined as a transducer that detects physical or chemical stimuli from the environment and converts them into quantitative electrical signals. From the perspective of mimicking human sensory systems, sensors can be broadly classified based on the type of stimuli they perceive. This classification includes (1) optical sensors for vision, (2) mechanical sensors encompassing tactile and auditory capabilities, and (3) chemical sensors that emulate olfactory and gustatory functions. Conventional sensing systems typically operate by periodically sampling all incoming analog signals and converting them into the digital domain. However, this approach has a fundamental limitation because the system indiscriminately collects not only meaningful information but also static background noise and redundant data. The high power consumption of ADCs required to process this massive volume of unstructured data along with the waste of data transmission bandwidth poses a significant challenge in the IoT era. In contrast, biological sensory systems maximize energy efficiency through mechanisms such as sensory adaptation, which suppress static background signals and selectively transmit only significant changes or events. Inspired by this biological efficiency, recent research has focused on next‐generation sensor technologies capable of filtering or pre‐processing redundant data at the sensing stage. This section focuses on the fundamental operating principles of sensor devices that serve as the foundation for such bio‐inspired systems. Specifically, Section 3.1 discusses the principles of optical sensors for light detection, while Section 3.2 covers mechanical sensors for perceiving pressure and sound. Finally, Section 3.3 discusses the operating principles of chemical sensors for gas and ion detection.

### 3.1 Optical Sensors (Vision Sensors)

Optical sensors function as transducers that convert external light energy into detectable electrical signals. This conversion process is broadly categorized into two main mechanisms based on the physical interaction between light and the material. The first is the optical excitation mechanism, where photons directly excite electrons, and the second is the thermal excitation mechanism, which utilizes heat generated from light absorption [151].

**FIGURE 3:**
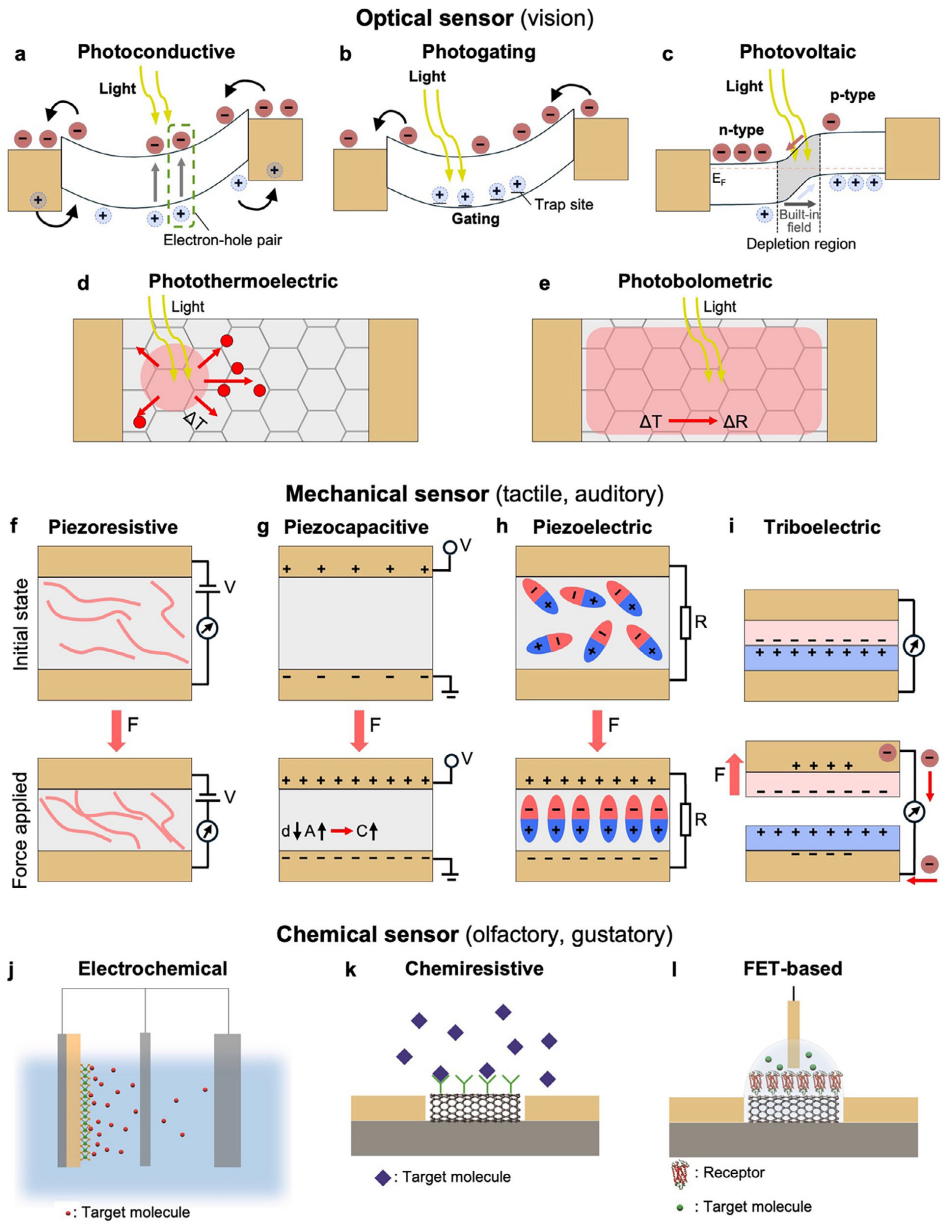
Working mechanisms of optical, mechanical, and chemical sensors. (a–e) Optical sensors categorized by excitation modes: optical excitation mechanisms include (a) photoconductive, (b) photogating, and (c) photovoltaic effects, while thermal excitation mechanisms include (d) photothermoelectric and (e) photobolometric effects. (f–i) Mechanical sensors operate via (f) piezoresistive, (g) piezocapacitive, (h) piezoelectric, and (i) triboelectric mechanisms. (j–l) Chemical sensors are based on (j) electrochemical, (k) chemiresistive, and (l) FET‐based detection.

The optical excitation mechanism relies on the generation of electron‐hole pairs when the energy of incident photons exceeds the bandgap of the semiconductor [152]. Depending on the behavior of charge carriers, this category is divided into photoconductive, photogating and photovoltaic effects [153]. The photoconductive effect refers to the phenomenon where electron‐hole pairs generated by photons with energy greater than the bandgap are excited to the conduction and valence bands, respectively, to become free carriers that move under the external electric field to increase the total current (Figure 3a) [154, 155, 156]. The photocurrent (*I*_ph_) is defined as the difference between the current under illumination (*I*_light_) and the dark current (*I*_dark_). To maximize the photoresponsivity based on photoconductive mechanism, avalanche photodiodes beyond typical optical sensors have also been used [157]. These devices operate near the breakdown voltage and utilize the impact ionization process where photocarriers generated by light acquire large kinetic energy from the strong electric field and collide with atoms. These collisions ionize the atoms and release secondary electrons, which are then accelerated to collide with additional atoms, causing an exponential increase in the electron count through an internal amplification mechanism [158]. This process enables large photocurrents and high signal‐to‐noise ratios. The photoconductive effect necessarily requires an external bias voltage, presenting a disadvantage in power consumption. However, operation within a single semiconductor material is possible, while using heterostructure architectures can facilitate carrier separation to induce faster response speeds [159, 160].

The photogating effect, which is a special case of the photoconductive effect, occurs when either an electron or a hole generated by a photon is trapped at a defect site within the semiconductor material [151]. The charged trap site acts as a localized floating gate that forms a photo‐induced shift in threshold voltage and strongly modulates the conductivity of the channel (Figure 3b) [161]. Notably, until the trapped charge recombines, the free carriers can recirculate through the channel multiple times, resulting in a very high photogain (*G*) [162]. This mechanism offers high sensitivity due to the long lifetime of trapped carriers but suffers from a slow response time caused by the slow rate of carrier detrapping [163]. An additional advantage is that photogating occurs at trap levels within the energy bandgap, allowing for the detection of sub‐bandgap light with energy smaller than the semiconductor bandgap [164].

Unlike the previous two effects that require external voltage, the photovoltaic effect generates an electromotive force by separating electron‐hole pairs through a built‐in electric field [165], which is formed at a *p*–*n* junction in a diode or a *p*–*n* heterostructure interface in a transistor (Figure 3c) [166, 167, 168]. Furthermore, aside from *p*–*n* junctions, an internal electric field can be induced by exploiting the work‐function difference between asymmetric electrodes contacting the semiconductor [161, 169, 170]. The separated electrons and holes move toward the *n*‐region and p‐region boundaries, respectively, and accumulate to generate a current allowing the device to operate without applied voltage or under reverse bias without an external bias, while exhibiting low dark current and high energy efficiency [151].

In contrast to optical excitation, the thermal excitation mechanisms operate by converting absorbed light into heat, which subsequently induces an electrical signal. The photothermoelectric (PTE) effect occurs through the combination of photothermal conversion and the thermoelectric effect [171]. When localized incident light irradiates the device, the temperature of the illuminated area rises, causing a local temperature difference within the device, which drives the diffusion of carriers from the high‐temperature region to the low‐temperature region (Figure 3d) [151]. Consequently, a voltage difference is induced between regions with different Seebeck coefficients due to the Seebeck effect [172]. This phenomenon is dominant in materials with low heat capacity such as graphene and black phosphorus (BP), where photogenerated carriers heat up rapidly before energy dissipates to the lattice [173, 174]. While sensors based on PTE can operate without an external bias, the induced voltage is typically very small requiring ohmic contacts at the metal/semiconductor interface for observation. The key advantage of PTE devices is the broadband response ranging from ultraviolet to far infrared, as the mechanism is not limited by the bandgap of materials [175]. However, compared to photoelectric devices, they typically exhibit slower response speeds due to the time required for the thermal transport process.

Finally, the photobolometric effect is based on the principle that the heating effect from photon absorption raises the temperature of the channel material, which changes the carrier mobility or carrier concentration, thereby inducing a resistance change (Figure 3e) [152]. Since light induces a change in resistance, the current increases or decreases compared to the dark state when a constant voltage is applied across the channel. Unlike the PTE effect, which is self‐driven and generates its own current, the bolometric effect relies on resistance change and thus functions only when an external voltage is applied [176, 177]. Materials with a high temperature coefficient of resistance (TCR), such as VO*_x_* and PdSe_2_, are advantageous for this sensing mechanism, which also allows for broadband response but is characterized by a slow response speed typically in the range of 1 ms to 100 ms [178, 179, 180, 181, 182].

### 3.2 Mechanical Sensors (Tactile and Auditory Sensors)

Mechanical sensors are devices that convert mechanical stimuli such as external pressure, vibration, and sound into electrical signals and are classified into piezoresistive, capacitive, piezoelectric, and triboelectric types based on their transduction mechanisms.

The most representative type is the piezoresistive sensor, which detects changes in electrical resistance caused by the deformation of a conductor or semiconductor material under external pressure (Figure 3f). This deformation induces changes in internal conductive pathways within the active layer, such as particle spacing, contact area, or tunneling resistance [183, 184, 185]. In conventional silicon‐based piezoresistive sensors, pressure‐induced deformation of silicon alters the crystal potential distribution, leading to a change in the electronic band structure and subsequently modifying carrier mobility to vary resistance [186]. In contrast, sensors based on metal–polymer composites (e.g., metal films or metal nanowires embedded in polymers) operate through a geometric effect, in which resistance varies with changes in length and cross‐sectional area under pressure [187]. Recent research on nanocomposites using polymers and carbon nanotubes (CNTs) has demonstrated that inducing changes in tunneling resistance and conductive paths in addition to lattice deformation and geometric effects can achieve higher sensitivity [188, 189]. Piezoresistive sensors offer high resolution, sensitivity, and linearity for both static and dynamic external stresses [190].

In contrast to piezoresistive sensors, capacitive sensors convert external pressure into a change in capacitance, which serves as the electrical signal. Capacitive sensors typically employ a parallel‐plate capacitor structure. When sound vibration or external pressure is applied, the sensor detects capacitance changes caused by a reduction in the distance between electrodes, an increase in contact area, or a change in the dielectric constant of the dielectric material (Figure 3g) [191, 192]. Based on the capacitance formula (*C*  = ε_0_ ε_*r*_ *A*/*d*), enhancement strategies target increasing the dielectric constant (*ε*_r_) through material design or tuning the electrode area (*A*) and separation distance (*d*) through structural engineering [193]. Recent research trends focus on using porous nanocomposites incorporating CNTs or graphene into polymers to enhance dielectric properties or developing flexible piezocapacitive sensors using polymer‐based materials [194, 195, 196, 197, 198]. Structurally, microstructuring techniques such as introducing wrinkles, pyramid structures, or half‐sphere structures are employed to increase sensitivity and achieve high sensing precision and flexibility [199, 200, 201, 202]. Capacitive sensors offer advantages such as low power consumption and high sensitivity; however, parasitic capacitance can introduce interference that acts as noise [191].

Unlike the previous two methods that require an external power source, the piezoelectric sensor is characterized by its ability to be self‐powered, generating its own voltage through internal polarization when subjected to pressure [192]. This mechanism is based on the direct piezoelectric effect, where mechanical pressure or vibration causes a displacement of ions within the crystal structure, leading to a change in the electric dipole moment (Figure 3h) [191, 203]. To counteract this change, charges of opposite polarity accumulate on the electrode surface, and the resulting potential difference is detected as a signal. While this method offers the advantage of fast response to instantaneous signals from dynamic pressure, the accumulated charge dissipates quickly due to circuit impedance, necessitating additional circuits to amplify the signal such as charge amplifiers [204, 205]. Furthermore, because the generated charge flows into the circuit and dissipates under constant pressure, reliable detection of static pressure is challenging, in contrast to piezoresistive and capacitive sensors that can continuously monitor static pressure. Representative materials with high piezoelectric charge coefficients include aluminum nitride (AlN) [206, 207], polyvinylidene fluoride (PVDF) [208, 209, 210, 211], and zinc oxide (ZnO) [212].

Finally, triboelectric sensors operate on the principle derived from the triboelectric nanogenerator (TENG), which converts mechanical signals into electrical signals through a combination of contact electrification and electrostatic induction (Figure 3i) [213, 214]. In contact electrification, when two different materials come into contact, electrons transfer from one to the other due to differences in electron affinity. When the materials separate, one surface remains positively charged, while the other remains negatively charged. Subsequently, as the two materials move apart or come closer, electrostatic induction drives the movement of charges through the electrodes connected to the back of the materials to balance the surface charges, thereby generating a voltage signal in the external circuit. To induce triboelectricity, materials with high electronegativity, such as PDMS [215, 216] or fluorinated ethylene propylene (FEP) [217, 218] are typically used as the negatively charged layer, while metals like Cu [219] and Al [220] or polymers like nylon [221] and polyurethane [222] are used as the positively charged layer. Like piezoelectric sensors, triboelectric devices have the advantage of being self‐powered and generates an alternating current (AC) signal, in which the current direction changes depending on the direction of mechanical motion [191]. Additionally, a major advantage lies in a broader range of material choices compared to other operating mechanisms [222, 223].

### 3.3 Chemical Sensors (Olfactory and Gustatory Sensors)

Chemical sensors are devices that convert the reaction with specific chemical substances into electrical signals and are classified into electrochemical, chemiresistive, and FET‐based types according to their operating principles.

First, electrochemical sensors analyze substances qualitatively and quantitatively by utilizing changes in electrochemical properties, such as potential, current, or impedance, resulting from the interaction between the electrode and substances in the solution (Figure 3j) [224]. These devices typically employ a three‐electrode system consisting of a working electrode, a counter electrode, and a reference electrode and are widely used as taste sensors because electrochemical sensing primarily targets analytes in the liquid state. The operating principles involve monitoring changes in charge, conductivity, or redox current generated when a biosensing material immobilized on the working electrode reacts with target molecules, thereby converting chemical information into electrical signals. Detailed measurement methods are broadly categorized into potentiometry, amperometry, and voltammetry [225]. In potentiometry, the working electrode is wrapped in a membrane, and upon contact with the electrolyte, a chemical reaction occurs at the surface, generating an electrical potential that is measured as a potential difference relative to the reference electrode. This voltage is measured in an equilibrium state where the net current is zero [226]. Voltammetry is a method of applying a time‐varying potential to the working electrode and measuring the resulting current [227]. When a varying voltage is applied to the working electrode, oxidation or reduction reactions of the analyte occur at the electrode interface, generating a current that is measured to identify the type and concentration of the substance [228]. Depending on the waveform of the applied voltage, the method is classified into cyclic voltammetry, which employs a triangular waveform, and pulse voltammetry, which applies periodic pulses while linearly scanning the voltage [229]. Amperometry involves applying a constant voltage and measuring the change in current overtime as the analyte is reduced or oxidized by the voltage [225]. Since the applied voltage must be set to a value sufficient to oxidize or reduce the analyte, voltammetry often serves as a preliminary step to determine the optimal conditions for amperometry [225]. In both voltammetry and amperometry, the generated current increases linearly as the concentration of the electrolyte or analyte increases. These electrochemical sensors have the advantages of using simple instruments and offering high sensitivity and fast response, but have the disadvantage of biofouling where the electrode surface becomes contaminated [225].

Next, a chemiresistive sensor quantitatively detects the type and concentration of substances by measuring the changes in electrical resistance that occur when gas‐ or liquid‐phase species contact the sensing material and is commonly used for olfactory sensors (Figure 3k) [224, 230]. When target molecules are adsorbed onto the active layer of the sensing material, physical and chemical reactions occur, leading to changes in electrical properties, such as charge carrier concentration and mobility, within the sensing material, thereby inducing a change in resistance. For instance, when an *n*‐type semiconductor is used as the sensing material, reacting with an oxidizing gas increases the adsorbed oxygen ions causing the resistance to change, whereas reacting with a reducing gas causes the resistance to change in the opposite direction [231]. Major sensing materials include metal oxides like tin oxide (SnO_2_) [232, 233] and ZnO [234] as well as conducting polymers [235, 236]. Metal oxides are characterized by strong sensitivity and rapid response but have the disadvantage of requiring high‐temperature processing [232, 237]. On the other hand, conducting polymers offer low power consumption due to ambient temperature processing but are susceptible to humidity [238].

Finally, FET‐based sensors are chemical sensors widely used for both olfactory and gustatory functions. These devices detect analytes by monitoring changes in surface charge density and surface potential as electrical signals when specific chemical species bind to the sensing layer or receptor (Figure 3l) [224]. Specifically, the analyte‐induced changes in surface charge and potential affect the density of charge carriers within the channel, thereby modulating the drain current or threshold voltage and amplifying minute chemical signals into electrical signals [239]. The device structure is a modification of the conventional MOSFET consisting of a semiconductor channel, a sensing layer, and an electrolyte gate. For the semiconductor channel, materials with large surface area, high conductivity, and high carrier mobility such as CNTs [240] and graphene [241] are used to enhance the sensitivity of charge transfer based on chemical reactions occurring through receptors [242]. The sensing layer is a receptor layer formed on the channel surface or gate insulator composed of protein‐based olfactory receptors or conducting polymers [243, 244]. In the case of the electrolyte gate, the analysis solution itself acts as the gate insulator or the gate voltage is applied through a reference electrode within the solution. Typically, by combining the device with human receptors, the change in charge generated during the chemical reaction between the receptors and reaction molecules induces a change in the channel current of the device [240, 241, 245, 246]. A major advantage of FET‐based chemical sensors is their compatibility with CMOS processes allowing for large‐scale integration on a single chip [247].

## 4 Architecture for Sensor‐Neuromorphic System Integration

Conventional sensing architectures typically adopt a physically separated approach in which sensing, data conversion, and digital processing are handled by different components. While this modularity has enabled rapid system development, the separation between components also amplifies the von Neumann bottleneck because substantial energy and latency are spent moving raw or lightly processed sensor outputs through interconnects and converters before reaching computing units. Neuromorphic sensors aim to alleviate this mismatch by producing computation‐ready representations (e.g., events or stateful signals) and/or by embedding synaptic/neuronal functions close to the sensing front‐end.

Two complementary directions have emerged. Near‐sensor processing reduces data‐movement cost mainly through system‐, circuit‐, and packaging‐level integration while keeping sensing and computation functionally separated. In‐sensor computing goes further by co‐localizing sensing and neuromorphic computation within a single device element, where stimuli directly modulate intrinsic state variables that encode synaptic/neuronal functions. From a functional perspective, the distinction between these two schemes lies in the stage of the information‐processing chain at which computation begins. In near‐sensor architectures, computation is introduced only after the sensor has completed stimulus transduction. By contrast, in in‐sensor architectures, computation begins during or immediately upon transduction, because the external stimulus directly updates a device‐embedded state variable that simultaneously serves sensing, memory, and computing functions.

In this Review, we classify sensor–neuromorphic system integration strategies primarily by architecture—the physical integration structure for near‐sensor approaches and the terminal configuration/vertical coupling for in‐sensor approaches—because these structural choices most directly determine the dominant bottlenecks (interconnect bandwidth/energy, conversion overhead, and area–performance trade‐offs) and enable consistent cross‐comparison across materials and platforms.

### 4.1 Near‐Sensor Processing: System‐Level Integration

**FIGURE 4:**
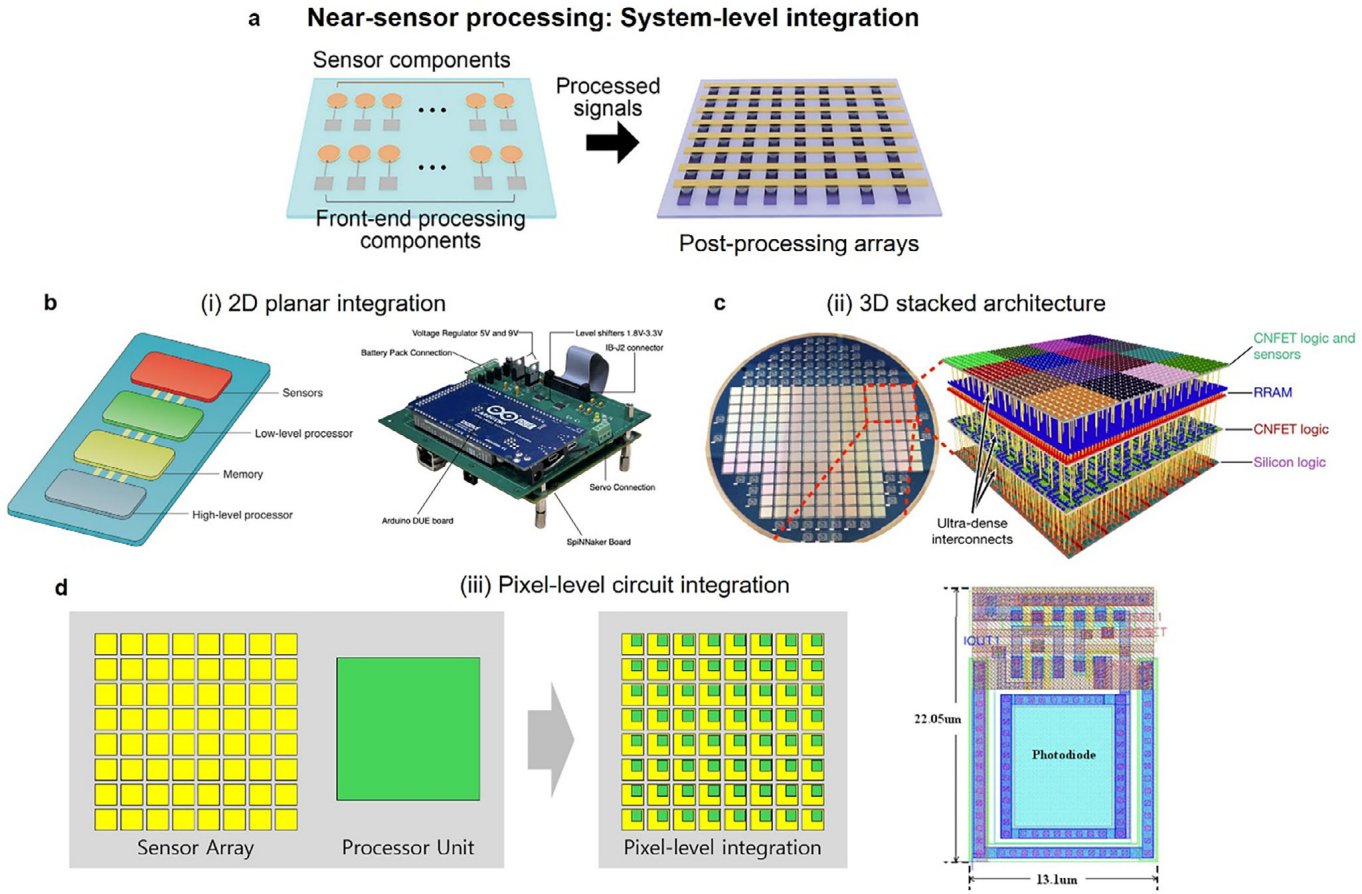
Architectures for near‐sensor neuromorphic integration. (a) Schematic of near‐sensor processing architecture for neuromorphic systems. Reproduced with permission from [248]. Copyright 2025, John Wiley and Sons. (b) Schematic illustration and practical example of board‐level 2D planar integration between the sensor and computing circuits. Reproduced (Adapted) with permission from [4, 252]. Copyright 2020, Springer Nature, and Reproduced (Adapted) under terms of the CC‐BY license, Copyright 2023, The Authors, published by MDPI. (c) 3D stacked architecture enabling vertical integration of sensor, memory, and logic layers. Reproduced (Adapted) with permission from [261]. Copyright 2017, Springer Nature. (d) Pixel‐level circuit integration for near‐sensor computation. Reproduced (Adapted) under terms of the CC‐BY license [267]. Copyright 2009, The Authors, published by MDPI.

In near‐sensor processing, the sensor and the processing/learning hardware remain functionally decoupled. The sensor primarily performs signal acquisition, while dedicated computing and/or memory units execute feature extraction, inference, or learning (Figure 4a) [248]. Crucially, however, the interface between the two is engineered to reduce the cost of data movement. This is achieved through circuit design and packaging choices that shorten interconnect length, increase parallelism in data transfer, and reduce redundant data generation. Depending on the integration scale, near‐sensor systems may operate at the board level (e.g., discrete sensor modules connected to CPUs/GPUs), at the chip level (e.g., vertically stacked dies with dense interconnects), or within pixel arrays that embed CMOS processing circuits. Across these architectures, the primary design axis is not whether the computation is neuromorphic in an algorithmic sense, but whether the architecture systematically collapses the distance between sensing and processing to improve bandwidth, latency, and energy efficiency. In this way, meaningful neuromorphic functionality can be demonstrated using largely conventional components through system‐ and circuit‐level co‐design.

#### 4.1.1 2D Planar Integration (Board‐Level)

The most accessible form of near‐sensor processing is 2D planar (board‐level) integration, where the sensor module and the computing/learning engine (e.g., CPU/GPU/FPGA/neuromorphic accelerator) are implemented as separate components on the same PCB (or across tightly coupled boards) and connected through conventional electrical interconnects (Figure 4b) [249, 250, 251, 252]. In this configuration, the sensing front‐end remains largely unchanged, and neuromorphic functionality can be demonstrated via system integration and firmware/software co‐design [253, 254]. Because of the reliance on mature components and standard assembly flows, board‐level integration is often the first practical step toward validating neuromorphic algorithms and end‐to‐end system concepts under realistic sensing conditions [255, 256].

Despite the clear advantages of board‐level planar integration for proof‐of‐concept demonstrations, the architecture remains fundamentally constrained by off‐sensor data transfer, including input/output (I/O) energy, bandwidth ceilings, and wiring‐induced latency [4, 257]. These limitations provide the primary rationale for progressing from board‐level prototypes to chip‐level vertical stacking and pixel‐level circuit integration, where the physical distance and interconnect bottlenecks can be reduced more aggressively.

#### 4.1.2 3D Stacked Architecture (Chip‐Level)

Moving beyond board‐level planar integration, 3D stacked architectures vertically integrate a sensor die with a logic and/or memory die, creating a chip‐level near‐sensor system in which inter‐die communication is no longer limited to the chip edge limit [258, 259]. Instead of relying on a small number of edge I/O pins and long PCB connections, stacked systems employ dense vertical interconnects to provide short, massively parallel signal pathways between the sensing layer and the processing/memory layer (Figure 4c) [260, 261]. This structural change enables data generated across the full sensor area to be transferred to the underlying electronics, improving effective bandwidth while maintaining a high sensing fill factor.

These features directly address the principal weaknesses of 2D board‐level integration: the dominance of off‐chip I/O energy, pin‐limited bandwidth, and wiring‐induced latency. By shortening the physical interconnect length from centimeters to micrometers and increasing the number of parallel links, 3D stacking can substantially reduce the energy required for communication and mitigate bottlenecks at system interfaces [262, 263]. Nevertheless, because sensing and computing remain functionally separated, sensory signals often still require routing into a logic layer and may involve digitization/quantization before downstream processing, thereby introducing remaining ADC/quantization overheads that motivate the transition toward pixel‐level circuit integration [264].

#### 4.1.3 Pixel‐Level Circuit Integration: Embedding CMOS Processing Units Within Pixel Arrays

Unlike board‐level planar integration or chip‐level 3D stacking—which primarily reduce the distance for data transfer while keeping most computation downstream—pixel‐level circuit integration brings processing directly into the pixel array [265]. By embedding CMOS processing units within each pixel, sensory signals can be conditioned and partially processed in the analog domain before they are digitized and exported (Figure 4d) [266, 267]. This enables early operations such as local accumulation [268], thresholding/event generation [269], simple filtering [270], or compressed readout [271], thereby reducing output data volume and relaxing ADC requirements while improving end‐to‐end latency and energy efficiency.

Structurally, this approach integrates a sensing element with dedicated in‐pixel circuitry such that the “weights” or internal states are stored as circuit variables rather than device‐state variables, offering a CMOS‐compatible route to near‐sensor neuromorphic prototypes (Figure 4d) [272]. Its limitations are clear: allocating area to computation reduces optical fill factor and can constrain spatial resolution, while dense mixed‐signal circuitry may increase design complexity and susceptibility to noise. These trade‐offs motivate the transition to device‐level in‐sensor computing, where sensing and state evolution are co‐localized without expanding pixel circuit area.

### 4.2 In‐Sensor Processing: Device‐Level Integration

**FIGURE 5:**
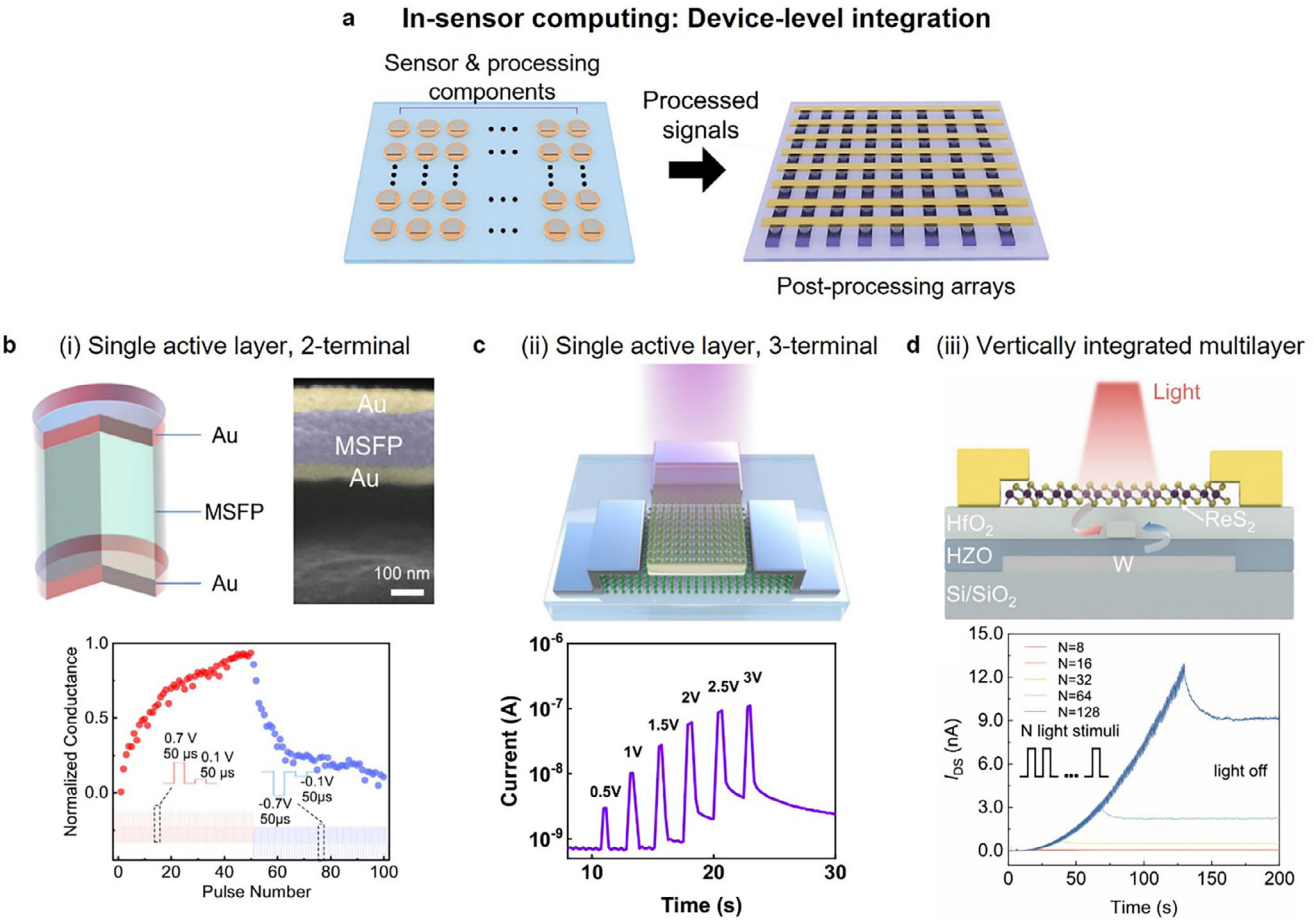
Architectures for in‐sensor neuromorphic integration. (a) Schematic of in‐sensor processing architecture for neuromorphic systems. Reproduced (Adapted) with permission from [248]. Copyright 2025, John Wiley and Sons. (b) Two‐terminal device with a single active layer and the corresponding analogue electrical resistive switching behavior. Reproduced (Adapted) under terms of the CC‐BY license [6]. Copyright 2023, The Authors, published by Springer Nature. (c) Three‐terminal device with a single active layer and corresponding channel current control through different pulse amplitudes. Reproduced (Adapted) under terms of the CC‐BY license [287]. Copyright 2024, The Authors, published by Springer Nature. (d) Vertically integrated multilayer optoelectronic synapse composed of a photosensitive ReS_2_ channel, a ferroelectric Hf_0.5_Zr_0.5_O_2_ layer, and a floating‐gate stack. Reproduced (Adapted) under terms of the CC‐BY‐NC‐ND license [295]. Copyright 2026, The Authors, published by Springer Nature.

As previously discussed, near‐sensor approaches mitigate the von Neumann bottleneck mainly by shortening interconnects or reducing data transmission volume. However, they still rely on functionally separated sensing and processing components, with remaining penalties tied to data transmission and digitization/quantization. In‐sensor computing addresses this limitation by performing sensing and neuromorphic computation within the same device element, without requiring additional external processing circuitry (Figure 5) [4, 273, 274]. In this paradigm, external stimuli (e.g., light, pressure, chemicals, or electrical pulses) directly modulate intrinsic device state variables—such as conductance, threshold voltage, polarization, or ionic/charge distribution—and the resulting state is retained over a relevant timescale [275]. Crucially, this state variable itself serves as the foundation for in‐sensor computation, encoding synaptic weight or neuronal excitability and enabling neuromorphic operations and learning directly at the sensing site [276, 277].

#### 4.2.1 2‐Terminal Devices With a Single Active Layer

Two‐terminal devices in which a single active layer simultaneously performs sensing and computation represent the most compact in‐sensor computing primitive, where external stimuli or electrical pulses directly tune an intrinsic state variable in a simple two‐terminal element (e.g., MIM‐type memristors [278], RRAM [279], or PCM [280]). The two‐terminal configuration enables a small cell footprint and is naturally compatible with crossbar‐array implementations, providing a practical route toward high‐density integration and parallel in‐memory/near‐sensor operations. Figure 5b demonstrates gradual, bidirectional conductance modulation in a two‐terminal device under pulse stimulation, enabling analog potentiation and depression as a hardware analogue of synaptic plasticity. The limitations of two‐terminal modulation emerge at the array level. In selector‐less crossbar arrays, undesired current paths through unselected cells (sneak‐path currents) can compromise read/write accuracy and increase energy consumption [281, 282, 283]. In addition, achieving highly linear and symmetric weight updates while minimizing read–write interference (disturbance) is often challenging, since the same terminals are typically used for both programming and sensing [284, 285]. These constraints motivate device‐ and circuit‐level mitigation strategies and provide context for three‐terminal gating approaches discussed next.

#### 4.2.2 3‐Terminal Devices With a Single Active Layer

Three‐terminal devices based on a single active layer implement in‐sensor computing in a gated‐device form, where a gate electrode provides an additional control degree of freedom over the active region. Figure 5c shows a three‐terminal multimodal photomemtransistor device exhibiting tunable synaptic plasticity, where both optical and gate‐pulse stimuli modulate the channel current and enable a transition from volatile short‐term responses (STM) to retained long‐term states (LTM) as the stimulus strength increases. In a three‐terminal configuration, external stimuli can modulate device‐embedded state variables such as threshold‐voltage shift, trapped/ionic charge distribution, polarization state, or channel doping state [286, 287, 288]. These physical states are retained or stably maintained under bias over a relevant timescale and manifest as changes in the transfer characteristics (*I*_d_–*V*_g_), thereby storing the synaptic weight within the single device element rather than in an external circuit state. In gated photodetector/photodiode arrays, the gate can similarly program the photo response (e.g., photoresponsivity), enabling synaptic weights to be encoded as a tunable photo response matrix [289, 290].

A key advantage of the third terminal is that the gate can decouple weight programming from readout/inference, improving tunability and often reducing read–write interference relative to two‐terminal operation [291, 292]. This added controllability can facilitate more gradual and symmetric weight updates, which is beneficial for learning accuracy at the array and system levels. A key limitation is materials‐level optimization. Within a single material platform, simultaneously optimizing high‐sensitivity stimulus transduction and long‐retention memory with low variability and low‐energy updates is difficult, motivating heterostructure‐based vertical integration strategies [293].

#### 4.2.3 Devices With Multiple Vertically Integrated Active Layers

To overcome the intrinsic trade‐offs of devices based on a single active material, stacking two or more functional active layers with complementary roles—typically a dedicated sensing layer and a dedicated plasticity/memory layer—has been investigated to realize multi‐functionality, which is difficult to achieve within a single material platform [294]. Importantly, this category is distinct from 3D stacked near‐sensor architectures: the goal here is not chip‐level packaging to shorten interconnects, but device‐level functional coupling between different materials, often through van der Waals stacking or thin‐film hetero‐integration, so that sensing and state evolution are co‐localized within a single vertically integrated element.

In vertically integrated multilayer neuromorphic devices, heterogeneous functional layers are stacked to unify optical transduction, state storage, and synaptic computation within a single compact architecture. For example, as illustrated in Figure 5d [295], a ReS_2_‐based metal–ferroelectric–metal–insulator–semiconductor ferroelectric field‐effect transistor integrates a photosensitive ReS_2_ channel with a ferroelectric Hf_0.5_Zr_0.5_O_2_ layer and a floating‐gate stack, enabling optical sensing, nonvolatile memory, and in situ computing in one vertically assembled element. Upon illumination, photocarriers generated in the ReS_2_ channel are translated into persistent conductance modulation through cooperative ferroelectric gating and floating‐gate‐assisted charge trapping, allowing optical inputs to directly program synaptic states. Moreover, the device exhibits characteristic synaptic plasticity, where increasing numbers of optical pulses enhance the excitatory postsynaptic current and extend its retention, reflecting a short‐term‐to‐long‐term memory transition. These results demonstrate that vertically integrated multilayer heterostructures provide an effective route to realizing compact optoelectronic synapses in which stimulus reception and memory‐state evolution are intrinsically linked.

For engineering‐oriented comparison, the major trade‐offs of representative near‐sensor and in‐sensor integration architectures are summarized qualitatively in Table 2. The representative architectures considered here include 2D planar integration [4, 251, 296], 3D stacked architecture [297, 298, 299], pixel‐level circuit integration [8, 248, 300, 301, 302], single‐active‐layer two‐terminal devices [6, 303], single‐active‐layer three‐terminal devices [248, 304, 305, 306], and vertically integrated multilayer devices [4, 295, 307, 308]. This comparison is intended as an architecture‐level guide rather than a strict quantitative benchmark, because the representative studies differ substantially in developmental maturity, implementation level, evaluation scope, and reporting practice, making direct numerical comparison potentially misleading.

**TABLE 2:** Qualitative comparison of key trade‐offs among representative near‐sensor and in‐sensor integration architectures. The architectures are compared in terms of energy efficiency, latency, fill factor, design complexity, scalability, and integration maturity. This table is intended as an architecture‐level summary for engineering‐oriented comparison rather than a strict quantitative benchmark [4, 6, 8, 248, 251, 295, 296, 300, 301, 302, 304, 305, 306, 307, 308].

Architectures/Metrics	Near‐sensor processing: System‐level integration			In‐sensor computing: Device‐level integration		
	2D planar integration	3D stacked architecture	Pixel‐level circuit integration	Single active layer 2‐terminal	Single active layer 3‐terminal	Vertically integrated multilayer
Energy efficiency	Low	Medium	High	High	Medium	High
Latency	Long	Medium	Short	Short	Short	Short
Fill factor	High	High	Low	High	Medium	High
Design complexity	Simple	Complex	Complex	Moderate	Moderate	Low
Scalability	High	Medium	Medium	High	Medium	Low
Integration maturity	High	Medium	Medium	Medium	Medium	Low

## 5 Bio‐Inspired Sensory Implementation

To effectively process massive amounts of data, recent research has increasingly focused on next‐generation sensor technologies capable of filtering or pre‐processing redundant data directly at the sensing stage, with both near‐sensor and in‐sensor strategies being actively explored. These neuromorphic systems exhibit fundamental differences in data processing compared to conventional von Neumann architectures. For dynamic vision, traditional frame‐based CMOS sensors synchronously digitize arrays at fixed frame rates, where maximizing the speed often amplifies noise and causes information loss. Conversely, event‐based neuromorphic vision sensors utilize asynchronous arrays where independent pixels generate spikes only upon detecting illumination changes, thereby minimizing data volume and power consumption while ensuring high temporal bandwidth [309, 310]. A similar paradigm shift is observed in artificial tactile systems. Conventional electronic skins rely on a serial data flow that transmits massive raw data to an external processor, causing severe energy overhead and data bottlenecks that limit their integration into edge devices. Neuromorphic tactile systems resolve this by generating spike‐based signals reflecting the temporal dynamics of mechanical stimuli, which enhances noise resistance and provides direct compatibility with neural computing frameworks [22, 311]. By emulating biological sensory adaptation mechanisms, including fast‐adapting (FA) and slow‐adapting (SA) properties, they effectively process complex spatiotemporal information, dramatically reducing the data‐processing burden for edge AI applications [312, 313].

Building on these neuromorphic principles across sensory modalities, we review recent research trends in bio‐inspired neuromorphic sensors, categorized into optical, mechanical, and chemical receptors based on the type of external stimuli they perceive. Following the near‐sensor and in‐sensor paradigms introduced in Section 4, we evaluate modality‐specific advancements in terms of their underlying neuromorphic mechanisms, physical integration structures, and terminal configuration/vertical coupling strategies. This approach allows for a systematic analysis of how diverse sensory inputs are efficiently processed within the structural constraints defined in the preceding section.

### 5.1 Neuromorphic Photoreceptors (Vision)

#### 5.1.1 Near‐Sensor Computing Systems

**FIGURE 6:**
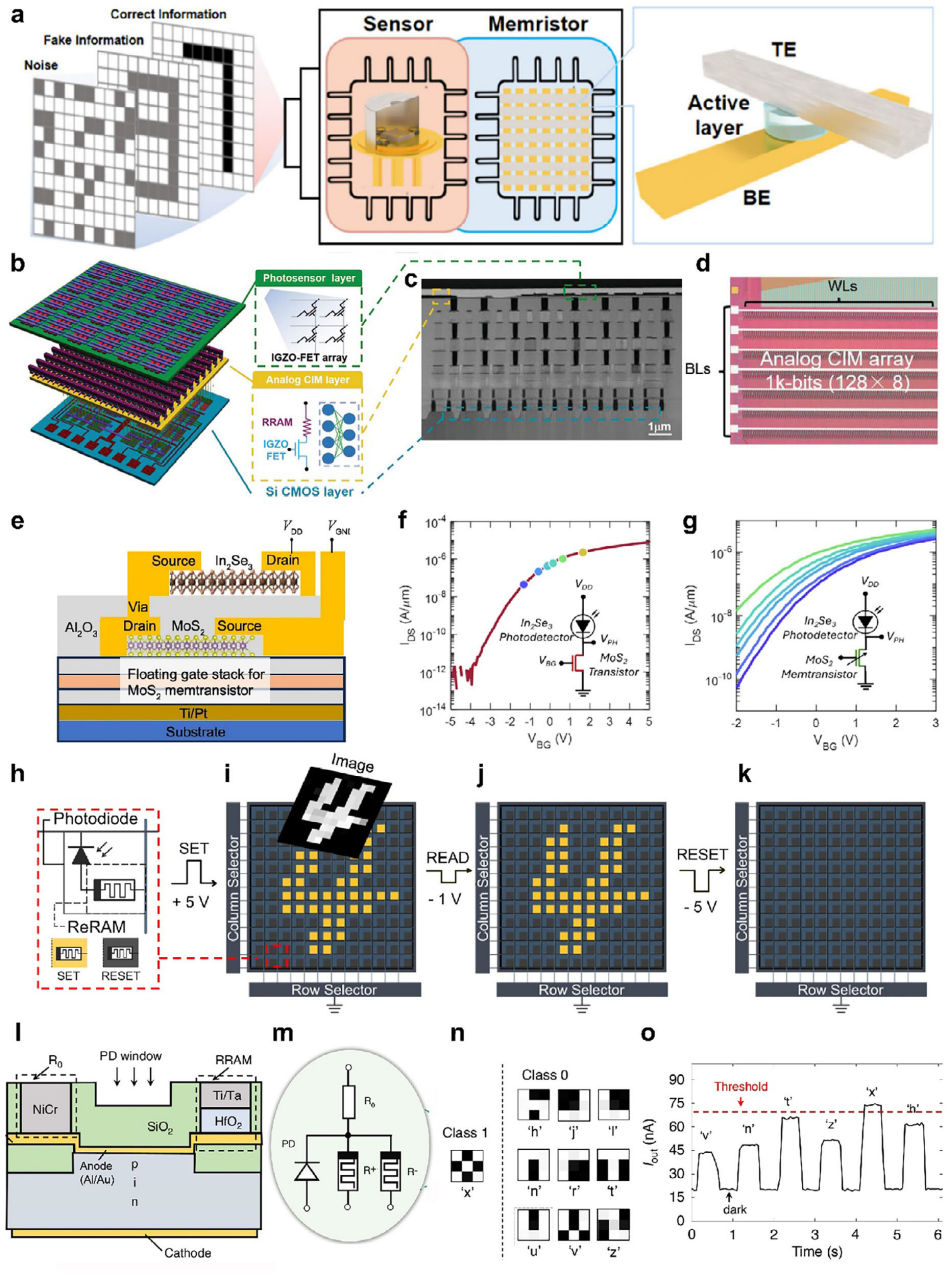
Neuromorphic photoreceptors based on near‐sensor integration. (a) The near‐sensor computing system with 2D planar integration architecture based on thermopile infrared detectors is capable of identifying light information in the infrared band from a mixture of light of various wavelengths and performing information storage and processing through the Pt/HfAlO_x_/TiN memristor. Reproduced (Adapted) with permission from [314]. Copyright 2025, American Chemical Society. (b) Schematic diagram of the 3D stacked architecture system consisting of three functional layers: the control logic layer, the analog computing‐in‐memory (CIM) layer, and the photosensor layer. (c) Cross‐sectional TEM image of the fabricated chip showing the three vertically stacked layers. (d) Top‐view microscope image of the 1 k‐bit analog RRAM/IGZO‐FET 1T1R array with 128 WLs and 8 BLs. Reproduced (Adapted) with permission from [319]. Copyright 2024, John Wiley and Sons. (e) Schematic of the 3D integrated In_2_Se_3_ photodetector and MoS_2_ memtransistor. (f) Different biasing states of the MoS_2_ transistor. (g) Different programmed conductance states of the MoS_2_ memtransistor. Reproduced (Adapted) under terms of the CC‐BY‐NC‐ND license [321]. Copyright 2025, The Authors, published by Springer Nature. (h) Circuit diagram for a pixel comprising a photodiode and a RRAM in the 16 × 16 1P‐1R crossbar array. The pixels are colored in yellow (blue) when the RRAMs are in a SET (RESET) state in (i–k). Schematic illustration of (i) image memorization, (j) readout, and (k) erasing processes in the 1P‐1R crossbar array. Reproduced (Adapted) under terms of the CC‐BY license [8]. Copyright 2022, The Authors, published by Springer Nature. (l) A single PD‐RRAM cell. Detecting mode: control terminals of the R0 and PD with the RRAM terminal floated. Writing mode: control terminals of the R0 and RRAM with the PD terminal floated. Reading mode: control three terminals under the short‐circuit condition. (m) Schematic illustration of the PD‐RRAM array utilizing pixel‐level circuit integration with a differential pair of RRAMs to realize signed weights. (n) Letter images of 3 × 3 pixels used for training/inference with 2‐class. (o) Measured output of the PD‐RRAM array for recognition. Projection of different letters with a duration of 0.5 s, leads to the distinct *I*_out_. Reproduced (Adapted) under terms of the CC‐BY license [315]. Copyright 2025, The Authors, published by Springer Nature.

Driven by energy and latency challenges from the AI and IoT data surge, bio‐inspired neuromorphic machine vision has emerged as a critical solution for advanced robotics and digital twins [314, 315, 316]. Near‐sensor architectures offer a promising solution to data bottlenecks by minimizing redundant transfer between sensing and computation [4]. For instance, Wang et al. integrate a photothermoelectric‐based infrared detector and a VCM‐based Pt/HfAlO*_x_*/TiN memristor in a 2D planar configuration (Figure 6a) [314, 317]. The electrical response generated by infrared signals inside the sensor serves as an input signal to generate action potentials that drive the memristor without requiring additional conversion circuits. This integrated system exhibits a wide temperature response range (100°C–300°C) and demonstrates the potential for noise removal, recognition, and encryption of infrared visual information in complex environments with various wavelengths of light. Specifically, the input voltage pulses derived from the infrared detector modulate the conductance of the memristor through the migration of oxygen vacancies and the dynamic formation and rupture of conductive filaments in the HfAlO*_x_* active layer. This physical process successfully replicates biological synaptic plasticity, generating distinct excitatory and inhibitory postsynaptic currents (EPSC and IPSC) in response to positive and negative stimuli. Furthermore, continuous sensory‐driven pulses induce progressive conductance changes, reliably emulating LTP and LTD behaviors with exceptional endurance and retention stability. By translating selective infrared sensory inputs into these stable synaptic weight updates, the integrated array can be effectively utilized for advanced visual information encryption, exclusively identifying target data hidden within complex environments.

To further minimize transmission latency and energy consumption through parallel data transfer, while achieving higher bandwidth and fill factors than 2D planar systems, research has shifted toward 3D integrated structures where photosensor and neuromorphic layers are vertically stacked [4, 318]. Figure 6b,c illustrates a monolithic 3D integrated (M3D) structure stacking a photogating‐based IGZO‐FET photosensor array, a 1T‐1R analog VCM‐based HfO_2_/TaO*_x_* RRAM/IGZO‐FET compute‐in‐memory (CIM) array, and Si‐based CMOS logic circuits [319]. Among these layers, the CIM structure implementing neuromorphic characteristics integrates an RRAM synaptic device, an IGZO‐FET controlling the RRAM current, and a crossbar with 128 word lines (WL) and 8 bit lines (BL) (Figure 6d). Serving as a thermal enhanced layer (TEL), the TaO*_x_* layer enables precise control over oxygen vacancy migration, allowing for the modulation of filament thickness and density to achieve 32‐level (5‐bit) analog weights. System simulations based on the M3D show 31.5 times lower energy consumption and 1.91 times faster operation compared to 2D planar integration structures, with an inference accuracy of 96.7%—comparable to a GPU. By achieving GPU‐level accuracy with a fraction of the energy cost, this architecture presents a highly promising pathway toward sustainable green computing in data‐intensive sensory networks.

To maximize energy efficiency and minimize latency, current research has shifted toward advanced pixel‐level array technologies, directly co‐locating the sensing element and synaptic/neuronal functional units within a single compact pixel [320]. This method is often referred to as “in‐sensor computing” as sensing and information processing occur simultaneously within a single pixel. However, in this Review, we categorize such approach as “near‐sensor computing” based on the structural feature of combining two physically distinct devices (sensor and computing unit). For example, Chowdhury et al. realize temporal optical signal processing with high accuracy in edge intelligence environments using an optoelectronic reservoir computing system that integrates an In_2_Se_3_ photodetector and a charge trapping/detrapping‐based MoS_2_ memtransistor monolithically integrated at the pixel level (Figure 6e) [321]. In this architecture, the natural variation in photoresponsivity—arising from the random thickness differences of the In_2_Se_3_ flakes—is actively exploited to enrich the reservoir states. However, handling such inherent device‐to‐device variations introduces significant calibration burdens in conventional readout circuitry. Figure 6f illustrates a conventional photoresponse calibration approach using an In_2_Se_3_ photodetector and a standard MoS_2_ transistor. In this setup, achieving optimal load resistance matching requires continuously adjusting the back‐gate voltage for every single pixel in real‐time. The individual modulation creates severe complexity and scalability issues in large arrays. In contrast, Figure 6g demonstrates a more efficient approach using MoS_2_ memtransistors with Al_2_O_3_/HfO_2_/Al_2_O_3_ floating gates. Because memtransistors are non‐volatile, the optimal conductance value for each pixel can be programmed and stored in advance. Consequently, a single, uniform voltage can be applied across the entire array during operation. This pre‐programmed weighting effectively compensates for device variations while dramatically simplifying the required circuitry. By converting daily stock index trends into 5 s pulse time series and training them with 6 pairs of In_2_Se_3_/MoS_2_ devices, the system achieves a prediction accuracy of *R* ^2^ = 0.88, demonstrating the robustness and efficiency of reservoir computing systems in processing high‐dimensional time‐series data with minimal hardware and training.

In another study, Lee et al. integrate an InGaAs *p*‐*i*‐*n* photodiode and an HfO_2_ RRAM into a 1‐photodiode and 1‐memristor (1P‐1R) crossbar array, demonstrating direct image memorization and in‐memory vector‐matrix multiplication for image encoding [315]. Figure 6h–k illustrates the image storage, reading, and deletion processes using a 16 × 16 1P‐1R focal plane array. Figure 6h shows the unit pixel circuit diagram, where yellow and blue pixels represent the LRS and HRS, respectively. During image storage, a +5 V voltage pulse (100 µs pulse width) is applied to each pixel (Figure 6i). The photodiode in the illuminated area becomes reverse‐biased, generating a photocurrent that forms a conductive filament in the RRAM, storing the information in an LRS. Stored images are read by applying a −1 V voltage pulse to each pixel (Figure 6j). At this point, the photodiode is forward‐biased and exhibits a very low resistance (<50 Ω) compared to the resistance range of RRAM (>10^3^ Ω). Thus, the 1P‐1R circuit can be approximated as a single RRAM circuit, and image data can be read without distortion by measuring the current flow. Finally, to erase the stored image, a −5 V voltage pulse (100 µs pulse width) is applied to each pixel to rupture the conductive filaments in the RRAM, resetting all pixels to the initial HRS (Figure 6k). Unlike other neuromorphic vision processing methods, the integrated system performs multiply‐accumulate (MAC) operations directly within the sensor by applying input voltages to the crossbar array instead of storing learned weights in memristors.

Research in neuromorphic vision is progressing from low‐level sensory processing toward hardware‐driven high‐level cognition. Inspired by the adaptive and selective nature of biological vision, researchers are now developing these complex tasks—such as real‐time object recognition—directly on neuromorphic hardware arrays, transforming sensors from simple imaging devices into autonomous recognition systems [8, 318]. Pan et al. integrate a Si *p*‐*i*‐*n* photodiode (PD) and RRAM into a single cell, realizing multi‐level photoelectric responses controlled by the non‐volatile and multi‐resistance state characteristics of the RRAM (Figure 6l) [8]. To increase the ratio of output current to dark current, a Ta/Ti/HfO_2_/SiO_2_/Au structure using switching layers of different materials is designed. To express signed weights, a pair of RRAMs was used in the unit cell to enhance system robustness (Figure 6m). The MAC operation capability between optical images and weights was experimentally verified using a PD‐RRAM array connecting multiple individual cells, and real‐time pattern recognition of optoelectronic signals was performed with a 3 × 3 array. Figure 6n shows the set of 3 × 3 pixel character images used in the training process. The target character “X” was classified as Class 1 and others as Class 0, trained as classifiers using positive weights. The weight matrix was then transferred to the PD‐RRAM array to program the theoretical resistance states into the RRAM for inference. During inference, images are projected onto the 3 × 3 PD‐RRAM array chip, where the PD generates photocurrent, and the RRAM performs MAC operations on the photocurrent to output recognition results via *I*_out_. Figure 6o shows the measured *I*_out_ when different characters are projected for 0.5 s. Experimental results demonstrate that when the target character (“X”) is presented, the output exceeds the threshold (70 nA), whereas the output remains below the threshold for other images, demonstrating excellent performance in image recognition with fast response and high SNR.

#### 5.1.2 In‐Sensor Computing Systems

**FIGURE 7:**
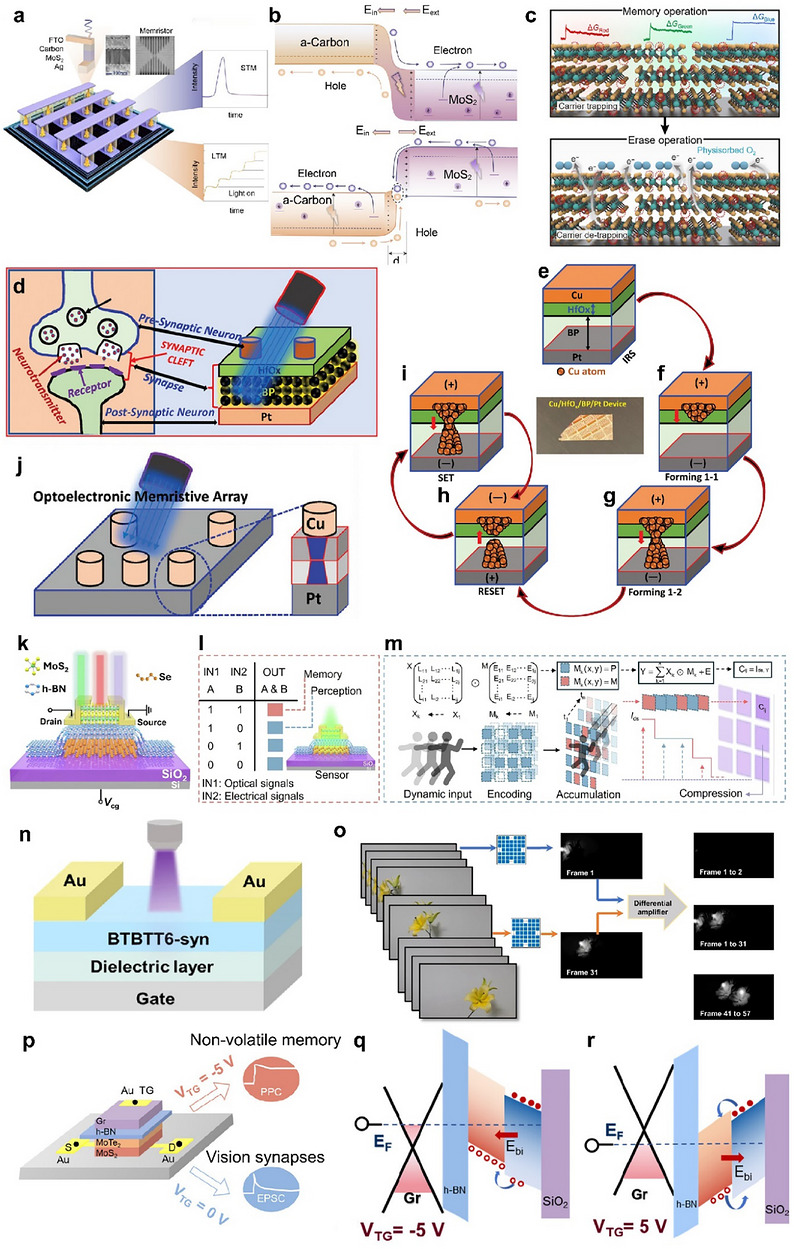
Neuromorphic photoreceptors based on in‐sensor integration. (a) A two‐terminal direct modulation optoelectronic memory array utilizing F‐doped SnO_2_/amorphous carbon‐MoS_2_/Ag heterostructures for reconfigurable short‐ and long‐term memory and (b) optical switching mechanism of the two modes. Reproduced (Adapted) with permission from [324]. Copyright 2026, John Wiley and Sons. (c) Vacancy‐driven working principle of a two‐terminal solution‐processed MoS_2_ in‐sensor device, where photoexcited carriers are trapped/de‐trapped at intrinsic sulfur‐vacancy‐localized states to enable memory and oxygen‐assisted erase operations. Reproduced (Adapted) under terms of the CC‐BY‐NC license [326]. Copyright 2026, The Authors, published by John Wiley and Sons. (d) Schematic comparison between a biological synapse and the artificial Cu/HfO*_x_*/BP/Pt memristive device based on two‐terminal direct modulation. (e–i) The conductive filament model surrounding a central top‐view optical image of the device, where HfO*_x_* (20 nm) and BP (≈40 nm) serve as active layers with Cu and Pt as top and bottom electrodes, respectively. The model shows: (e) initial state, (f,g) forming process, (h) RESET process, and (i) SET process. (j) The optoelectronic memristive array capable of sensing and processing data for the neuromorphic vision system. Reproduced (Adapted) under terms of the CC‐BY license [325]. Copyright 2023, The Authors, published by John Wiley and Sons. (k) Schematic of a single Se/h‐BN/MoS_2_ optoelectronic memtransistor based on a 3‐terminal architecture with multiple vertically integrated active layers. (l) Optoelectronic AND logic gate operation for sensing and compressing. (m) Algorithm workflow for in‐sensor encoding and compression of dynamic images using the optoelectronic sensor array. The combined optical (*L_ij_*) and electrical (*E_ij_*) inputs process the *k*‐th frame (*X_k_*) alongside its corresponding mask (*M_k_*). The compressed pixel value (*C_ij_*) is directly mapped to the drain‐source current (*I*_ds_) of the device, generating the final compressed output matrix (*Y*). Reproduced (Adapted) under terms of the CC‐BY‐NC‐ND license [328]. Copyright 2025, The Authors, published by Springer Nature. (n) Schematic of the UV‐sensitive organic neuromorphic vision sensor based on a three‐terminal architecture with a single active layer. (o) Data processing flow for motion detection using the BTBTT6‐syn‐based synaptic organic phototransistor arrays. Reproduced (Adapted) under terms of the CC‐BY license [306]. Copyright 2023, The Authors, published by Springer Nature. (p) Functional demonstration of a 3‐terminal architecture with multiple vertically integrated active layers, combining sensing, storage, and computing in a single device. The operation modes are reconfigurable between photo‐sensor/memory and vision synapses by modulating the top gate voltage *V*_TG_. (q,r) Energy band diagrams of the device corresponding to the two modes. Reproduced (Adapted) with permission from [288]. Copyright 2025, John Wiley and Sons.

To mimic the hierarchical information processing of biological visual systems, recent research in in‐sensor computing has evolved toward intrinsic reconfigurability, leveraging device physics to flexibly switch between sensing and memory modes within a single device [318, 322, 323]. Two‐terminal conductive filament‐based optoelectronic memristors are gaining attention as optimal solutions for high‐density arrays and 3D vertical stacking compatible with CMOS BEOL processes due to their simple MIM structure [4]. The synergy of light and electric fields allows precise control of filament behavior to represent analog weights [279, 324, 325]. The flexibility to switch between STM and LTM within a single device provides an efficient hardware platform for next‐generation in‐sensor neuromorphic vision systems where sensing, memory, and computation are integrated. For example, Wang et al. demonstrate a two‐terminal optoelectronic memory array with an F‐doped SnO_2_/carbon/MoS_2_/Ag structure, achieving biomimetic in‐sensor computing through self‐adaptive switching between dynamic STM and non‐dynamic LTM modes (Figure 7a) [324]. Optoelectronic memory devices based on this carbon/MoS_2_ heterojunction can selectively switch modes by controlling the width of the depletion region at the interface according to the polarity of the applied bias (Figure 7b). When a negative bias is applied, a wide depletion region forms at the interface, limiting carrier movement and maintaining a low‐conductance state. Under optical stimulus (405 nm UV), electrons trapped in MoS_2_ defects are de‐trapped, temporarily increasing conductance. However, the electrons quickly recombine with traps due to the extended depletion region, resulting in relaxation‐based STM characteristics. Conversely, under positive bias, the depletion region narrows, allowing photogenerated electrons to move between *n*‐type MoS_2_ and *p*‐type carbon layers without obstruction. The efficient carrier transport suppresses charge recombination, resulting in non‐volatile LTM characteristics where high conductance is maintained after the stimulus is removed. In LTM mode, 128 photoconductance states significantly improve energy efficiency for low‐bit tasks such as edge detection with an ultra‐low consumption of 1.6 fJ for a single convolutional kernel operation. In STM mode, transient photocurrent responses under negative bias enable the classification of 26 characters with 100% accuracy via reservoir computing (RC). The synergy of optical pulse modulation and electrical pulse calibration maps optical input directly to conductance changes, eliminating analog‐to‐digital conversion bottlenecks.

Beyond heterostructure‐based 2‐terminal platforms, an alternative approach utilizing single‐material architectures that leverage intrinsic material defects has emerged as a viable route for in‐sensor computing. For instance, Kim et al. realize a scalable array of lateral 2‐terminal devices using solution‐processed MoS_2_ thin films. The solution‐processing naturally introduces chalcogen vacancies that serve as localized trap states within the MoS_2_ network. Under optical illumination, photoexcited carriers are trapped in these vacancy‐localized states (Figure 7c) [326], which enhances the photogating effect and markedly increases the device conductance. Due to the extended lifetime of the trapped charges, the photo‐induced conductance persists even after the cessation of the light stimulus, facilitating persistent photoconductivity (PPC) for localized memory operation. These programmed states can be subsequently reset through oxygen‐mediated de‐trapping process (Figure 7c). When exposed to an oxygen‐rich environment, physically adsorbed oxygen molecules function as strong electron acceptors. They effectively extract the trapped electrons from the localized states, thereby restoring the conductance toward the dark baseline. Furthermore, the wavelength‐dependent modulation of conductance (Δ*G*_Blue_>Δ*G*_Green_ >Δ*G*_Red_) enables direct, color‐selective weight encoding. As the optical pulse number increases, the inter‐color conductance contrast progressively enlarges. When integrated with a convolutional neural network framework, these encoded states enable a color recognition accuracy of up to 94%, ultimately providing a scalable pathway toward multi‐color retinomorphic systems without the necessity of complex heterojunction engineering.

To fully realize the potential of such retinomorphic systems and closely emulate the human retina in wearable platforms, recent research has been propelled toward flexible neuromorphic electronics. Kumar et al. propose a flexible, back‐end‐of‐line (BEOL) compatible two‐terminal optoelectronic memristor based on a solution‐processable black phosphorus (BP)/HfO*_x_* bilayer (Figure 7d) [325]. The Cu/HfO*_x_*/BP/Pt memristor exhibits ECM operation based on the electrochemical migration of Cu ions, with switching behavior determined by the differences in ion mobility and thermal conductivity between the HfO*_x_* and BP layers (Figure 7e–i). During the SET process, Cu ions from the top electrode pass through the high‐barrier HfO*_x_* layer and the high‐permeability BP layer (LRS). The variation in Cu ion transport rates between HfO*_x_* and BP layers results in an hourglass‐shaped conductive filament. Upon the RESET process, the high thermal conductivity of the BP layer (12 W m^−1^ K^−1^) facilitates filament rupture at a specific location, establishing the high‐resistance state (HRS). BP facilitates efficient heat dissipation toward the bottom electrode, shifting the Joule‐heating hotspot from the bottom contact to the HfO*_x_*/BP interface. Consequently, the filament ruptures occur locally at the thermally and structurally vulnerable heterojunction interface, demonstrating stable resistance‐based synaptic properties over 1000 epochs with 400 conduction pulses. These results suggest that the thermal properties of 2D materials are crucial variables in controlling memristor switching dynamics. The optoelectronic memristive array for neuromorphic vision systems (Figure 7j) maintains stable synaptic properties even when bent to a 1 cm radius using a silicon back‐etching process, proving its compatibility with back‐end processes and suitability as an optoelectronic memory storage device for platforms operating under rigorous mechanical conditions.

While two‐terminal optoelectronic devices offer advantages in high integration density and process simplicity, high dark current under high bias due to the lack of a control terminal (gate) remains a challenge [290, 318]. Increasing the applied voltage to achieve high photoresponsivity also increases dark current, degrading standby power and SNR, which limits the detection of weak optical signals. In contrast, three‐terminal devices consisting of source, drain, and gate electrodes resolve this issue by physically separating the sensing and control paths [315, 318, 327]. Particularly, electrostatic doping effects using gate voltage allow flexible adjustment of the operating mode of the device, providing a foundation for more complex and sophisticated synaptic plasticity within a single device. For instance, a 2D material‐based three‐terminal architecture with multiple vertically integrated active layers has been implemented to efficiently capture and compress multidimensional signals containing spectral and temporal information (Figure 7k) [328]. The 2D sensor consists of a photosensitive Se charge trapping layer, an h‐BN insulating layer that controls charge transfer, and a MoS_2_ channel layer. This configuration enables the sensor to capture, memorize, and encode optical signals, achieving an in‐device snapshot compression of dynamic video data with an 8:1 compression ratio. The device operates in two distinct modes depending on the applied stimuli. In the absence of a gate voltage, illumination generates electron‐hole pairs within the Se layer, reducing the contact potential difference between Se and MoS_2_. The resulting drop in contact potential induces a strong photogating effect in the MoS_2_ layer, allowing the device to operate in a volatile perception mode. When a gate voltage and an optical signal are applied simultaneously, the energy band bending of MoS_2_ drives carriers to tunnel through the h‐BN barrier and become trapped in the Se layer. The trapped charges shift the threshold voltage (*V*_th_) in a non‐volatile manner, encoding and storing the optical signal as a persistent change in MoS_2_ channel conductance. This process operates analogously to an optoelectronic AND logic gate, as recording occurs only when both optical and electrical signals are present simultaneously (Figure 7l). Furthermore, since the channel conductance is linearly modulated according to the incident light intensity or the number of pulses, various pixel values can be compressed and encoded into channel current values. Figure 7m illustrates the snapshot compressive imaging operation, which integrates sensing, encoding, and video compression within a single device. During the encoding process, the electrical and optical signals simultaneously co‐modulate the conductance only when both the pixel value of the binarized video, *X*_k_(*x*, *y*, *t*), and the corresponding mask, *M*_k_(*x*, *y*, *t*), are “1.” The pixel values, encoded as non‐volatile conductance changes, accumulate over time from frame *t*_1_ to *t*_n_. The final conductance represents the integrated and compressed pixel value, *C*, which is proportional to the number of simultaneous optical and electrical pulses. Subsequently, reading the unique current value determined by the synergistic action of the electro‐optical pulses allows for direct conversion into the pixel value of the compressed 2D image (*C* = *I*_ds,Y_). Finally, the compressed 2D image is reconstructed into a 3D data cube using the Plug‐and‐Play (PnP) algorithm. Collectively, these results establish the device as a highly miniaturized and effective 2D material‐based hardware encoder for video and spectral snapshot compressive imaging, demonstrating the strong potential of van der Waals heterostructures as a compact, energy‐efficient platform for future intelligent machine vision systems.

Beyond emulating human neural structures, recent research has further expanded into biomimetic systems inspired by the diverse visual capabilities found in nature. Jiang et al. realize a neuromorphic in‐sensor computing device specialized for the ultraviolet (UV) spectrum, inspired by the tetrachromatic vision system of butterflies, by utilizing phototransistors (OPTs) based on the asymmetric organic semiconductor material BTBTT6‐syn (Figure 7n) [306]. The ultra‐weak UV detection performance (31 nW/cm^2^) is attributed to a charge‐trapping mechanism driven by high‐density hydroxyl groups (‐OH) at the SiO_2_ dielectric interface. Analysis of interfacial energetic properties through the exciton binding energy reveals that the bare SiO_2_ interface exhibits a significantly higher exciton binding energy (*E*_B_) of 127.8 meV compared to polymer‐buffered interfaces (∼13–16 meV). The results paradoxically demonstrate that the superior sensitivity arises from a gating effect—prolonging hole lifetime via electron capture at interfacial traps—rather than spontaneous charge separation facilitated by low binding energy. Interfacial trap states serve as a critical mechanism for both signal amplification and the realization of synaptic plasticity (LTP/LTD) through charge retention. Consequently, the architecture effectively extracts ultraviolet information from color images to reduce redundant data, successfully demonstrating the dynamic motion detection of petals and pistils (Figure 7o).

To achieve reconfigurable synaptic functionalities, researchers are increasingly utilizing vertically integrated multilayer devices that can switch between different synaptic modes depending on the operational requirements. Zhao et al. introduce a multi‐mode three‐terminal heterojunction device based on a graphene/h‐BN/MoTe_2_/MoS_2_ architecture [288]. Beyond being a conventional three‐terminal transistor, the system is specifically defined as a vertically integrated multilayer device to highlight the synergistic integration of constituent layers with distinct roles: the MoS_2_ layer serves as the charge transport channel, while the MoTe_2_ layer acts as a sensitizing layer for light detection and charge storage positioned between the gate and the channel (Figure 7p). The operational mechanism centers on controlling the Fermi level of the ambipolar MoTe_2_ via the top‐gate voltage (*V*_TG_) to reconfigure the band alignment and internal electric field (*E*_in_) at the MoTe_2_/MoS_2_ interface (Figure 7q,r). Application of a negative voltage (−5 V) to *V*_TG_ induces *p*‐type behavior in the MoTe_2_, generating a robust vertical electric field that traps photogenerated holes within the floating MoTe_2_ layer. The resulting interaction triggers a strong photo‐gating effect in the MoS_2_ channel, yielding a high responsivity of 6.515 × 10^3^ A/W and non‐volatile persistent photoconductivity (PPC) behavior. Conversely, a positive voltage reverses the electric field direction, switching the device to a synaptic mode. The system‐level efficacy is verified through ANN simulations using experimentally extracted synaptic characteristics, which achieve an MNIST image classification accuracy of 95.26%.

### 5.2 Neuromorphic Mechanoreceptors (Tactile & Auditory)

#### 5.2.1 Near‐Sensor Computing Systems

**FIGURE 8:**
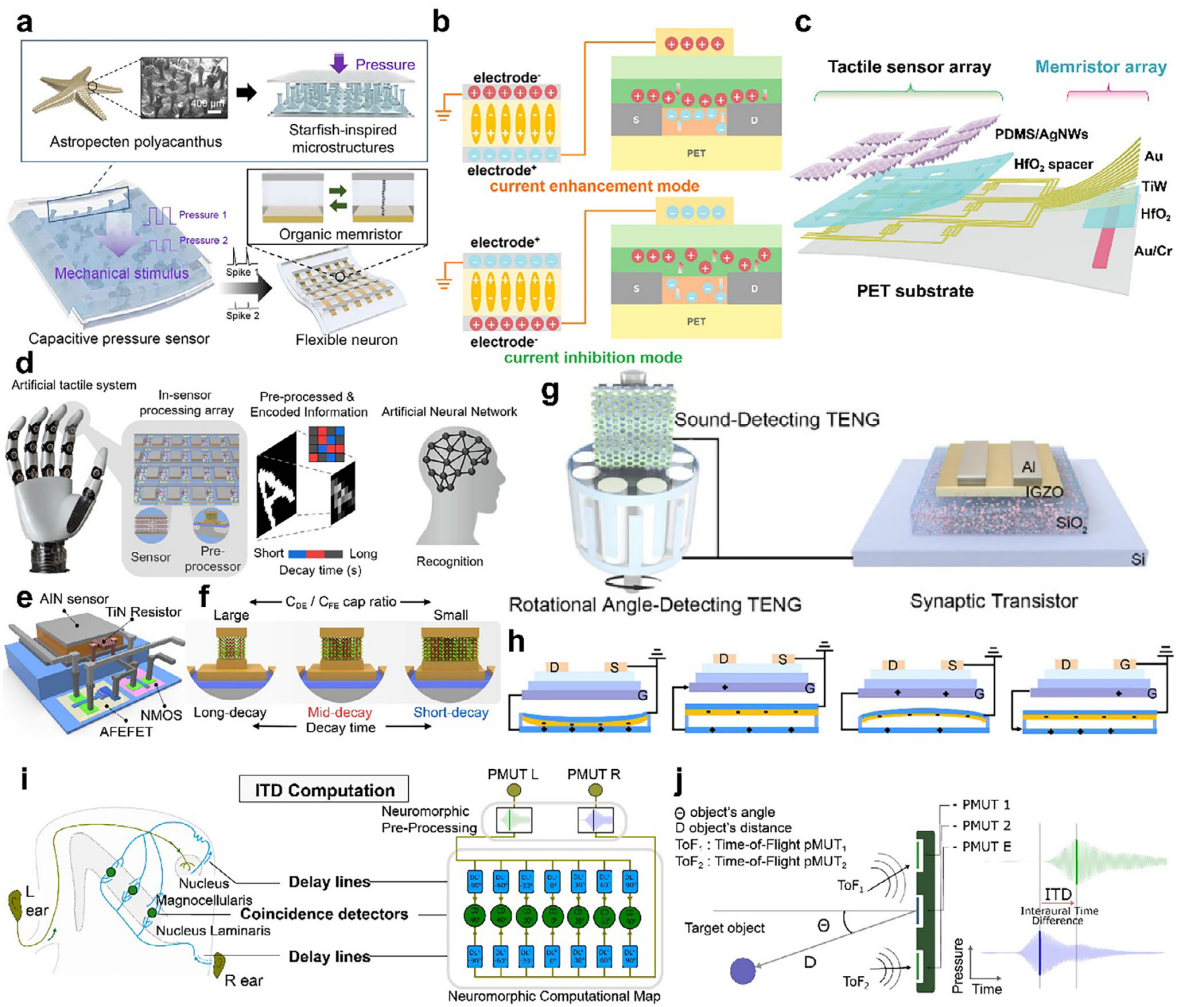
Neuromorphic mechanoreceptors based on near‐sensor integration. (a) Conceptual illustration of a tactile sensor system based on 2D planar integration, combining a capacitive sensor with a flexible memristor exhibiting synaptic plasticity. The device features a microstructured dielectric layer mimicking the surface of the starfish, Astropecten polyacanthus, designed for energy‐efficient near‐sensor computing. Reproduced (Adapted) under terms of the CC‐BY license [331]. Copyright 2025, The Authors, published by American Chemical Society. (b) Operation mechanism of the tactile‐stimulus modulated synaptic transistor based on the 2D planar integration of a piezoelectric sensor and a FET synaptic transistor. Reproduced (Adapted) with permission from [329]. Copyright 2025, American Chemical Society. (c) Flexible near‐sensor analog computing system utilizing a 3D stacked architecture capable of integrating a 3 × 3 pyramidal pressure sensor array and a 9 × 1memristor array. Reproduced (Adapted) with permission from [332]. Copyright 2022, John Wiley and Sons. (d) Schematic of the artificial tactile system featuring pixel‐level circuit integration, illustrating the flow from near‐sensor processing to the neural network for pre‐processing and recognition. Schematic diagrams of (e) the unit cell integrated with an AlN sensor and an AFEFET, and (f) the AFEFETs with various CDE:CFE ratios showing Long‐ (10:1), Mid‐ (5:1), and Short‐decay (2:1) behaviors. Reproduced (Adapted) under terms of the CC‐BY license [333]. Copyright 2025, The Authors, published by John Wiley and Sons. (g) Structural diagram of the bionic multisensory integration device and (h) the working mechanism and performance after the connection of the sound‐detecting TENG and the synaptic transistor. Reproduced (Adapted) with permission from [334]. Copyright 2025, Elsevier. (i) (left) The ITD computation model (Jeffress model) describes sound encoding in the nucleus magnocellularis (NM) and processing in the nucleus laminaris (NL). (right) Schematic of the neural ITD computational map utilizing 2D planar integration. This RRAM‐based neuromorphic architecture combines delay lines and coincidence detector neurons to model the owl's biological system. (j) Mechanism for object localization. A target object is located at an azimuthal position θ and distance D. An emitter pMUT produces a waveform at 117.6 kHz that is reflected by the object and arrives at the two pMUT receivers with different times‐of‐flight (ToF). This difference is defined as the interaural time difference (ITD) and serves as an indicator for the object's position. The ITD can be estimated by evaluating the peak of the response in the two receiver sensors. Reproduced (Adapted) under terms of the CC‐BY license [253]. Copyright 2025, The Authors, published by Springer Nature.

Near‐sensor computing emulating biological sensory processing enables immediate feedback for tactile stimuli and sophisticated sound localization mechanisms at the sensor level [253, 329, 330]. The approach resolves data bottlenecks to the central processor, achieving maximized energy efficiency and low‐latency real‐time performance for both delicate force‐controlled tactile tasks and complex spatial analysis in auditory tasks [331, 332, 333]. For example, Cho et al. realize an integrated system with bio‐equivalent energy efficiency by combining a flexible memristor with a capacitive pressure sensor based on an hourglass‐shaped microstructure that mimics starfish surfaces within a single pixel (Figure 8a) [331]. Upon pressure application, the transient potential generated during the charging of the capacitive sensor is transferred to the memristor and triggers the formation of Ag conductive filament through the ECM effect. The subsequent formation and regulation of Ag conductive filaments enable synaptic plasticity, encompassing both STP and LTP. By transforming mechanical stimuli into instantaneous electrical spikes instead of utilizing continuous DC voltage, the architecture fulfills artificial nociceptor functions while attaining an ultra‐low energy consumption of approximately 2.2 fJ per pixel, a performance level comparable to biological systems. This biological‐level energy efficiency underscores the potential of the near‐sensor system as a foundational technology for realizing sustainable green computing and reliable operation under mechanical conditions in future sensory networks.

In another study, Zeng et al. develop an artificial tactile perception system by serially combining a flexible PVDF piezoelectric sensor and a three‐terminal thin‐film transistor, mimicking recognition and response scenarios for pain and itch sensations using feedback control [329]. To enhance both the LTP and STP of the artificial synaptic transistor, a composite gate dielectric was developed using a ferroelectric terpolymer P(VDF–TrFE–CFE) and chitosan, while amorphous metal oxide InZnO was selected for the channel layer. The piezoelectric potential generated by the PVDF sensor under external pressure acts as the gate voltage to modulate channel conductance, allowing for the selective reconfiguration of synaptic operation modes by controlling the connection polarity between the PVDF sensor and the gate electrode (Figure 8b). In the current enhancement mode, where a positive dipole potential is applied to the gate, electrostatic induction promotes electron accumulation in the channel and interfacial proton migration within the chitosan electrolyte, generating an excitatory postsynaptic current (EPSC) for pain recognition. Conversely, the current inhibition mode applies a negative potential to deplete carriers and repel protons, inducing an inhibitory postsynaptic current (IPSC) to simulate itch sensations and their subsequent relief. The ferroelectric/electrolyte dual modulation mechanism successfully captures complex biological feedback loops by distinguishing between pain and itch‐relief functions based on the polarity‐dependent current response.

Other instances include the adoption of 3D vertically stacked structures designed to enable concurrent tactile signal input from neighboring pixels for spatial identification tasks like edge detection and texture recognition [332]. Figure 8c shows a synaptic device stacking a PDMS/Ag nanowire‐based piezoresistive pressure sensor array and an Au/TiW/HfO_2_/Au memristor array [332]. Application of external pressure expands the contact area between the pyramidal microstructures and electrodes, thereby decreasing the contact resistance. The analog resistance modulation is directly converted into input current for the memristor array without requiring a separate ADC circuit. The input signal integrates with the memristive conductance weights to physically execute VMM operations based on Ohm's law and Kirchhoff's current law. The architecture facilitates immediate sensor‐level image pre‐processing, including noise reduction and edge detection, achieving an ultra‐fast response speed on the order of 400 ns.

Jung et al. demonstrate a high‐efficiency in‐pixel integrated tactile perception platform by vertically stacking a piezoelectric AlN sensor and an antiferroelectric field‐effect transistor (AFEFET) within a single unit cell to maximize integration density and optimize signal transmission paths (Figure 8d,e) [333]. The AFEFET utilizes a metal‐ferroelectric‐metal‐insulator‐semiconductor (MFMIS) gate structure to enable reservoir computing capabilities, leveraging both spatial and temporal dynamics within a single device. Within this structure, the Zr‐rich leaky antiferroelectric HZO layer performs a spontaneous refresh function in which the stored charge is discharged on its own without a separate external voltage based on the property of near‐zero remnant polarization. The inherent transient response characteristics provide the STM capabilities, establishing this architecture as a physical reservoir for real‐time spatiotemporal tactile pattern recognition. Furthermore, structural control of the floating electrode area within the MFMIS framework (Figure 8f) modulates the capacitance ratio (*C*_DE_:*C*_FE_) between the ferroelectric and dielectric layers, which permits versatile tuning of discharge decay times and significantly expands spatiotemporal data processing capability. As a result, a spatiotemporal filter configuration leveraging AFEFETs with diverse decay dynamics achieves a 55.44% improvement in pattern recognition accuracy compared to a conventional filter where all devices maintain identical decay times.

Along with tactile sensing, emulating the human auditory system has also emerged as a key area of interest for implementing complex near‐sensor perception. The human inner ear serves as a sophisticated multisensory structure consisting of the cochlea for acoustic detection and the vestibular system for rotational sensing, and recent research aims to mimic the biological integration of such inputs through advanced electronic systems [334]. A biomimetic auditory‐rotatory integration device incorporates a PVDF‐based sound‐sensing TENG, a PTFE‐based rotation angle‐sensing TENG, and a Si/SiO_2_/IGZO/Al synaptic transistor in a 2D planar configuration (Figure 8g) [334]. The sound‐sensing TENG operates through periodic vibrations of a micropyramid array induced by sound pressure (Figure 8h). When the contact surface between the array and the lower electrode is separated by a change in sound pressure, a potential difference is generated, which causes positive charges to move to the gate of the synaptic transistor to balance the voltage. Conversely, when the array returns, the charges are returned to the TENG electrode. The periodic charge transfer driven by mechanical oscillatory motion generates the gate voltage for the synaptic transistor, facilitating self‐powered sensing and data processing. Simultaneously, the rotatory TENG detects rotational angles by generating triboelectric spike signals as a PTFE roller moves across electrodes due to inertial forces. Integration of the auditory and rotatory signals as gate voltages enables the generation of an EPSC. Exploiting the principle of temporal coincidence for multisensory integration, the combined voltage signals trigger the migration of protons (H^+^) within a porous SiO_2_ electrolyte toward the IGZO channel interface. The resulting formation of an EDL at the nanogap allows for high‐precision analog control of channel conductance. At the precise moment the sound sensor aligns with the actual sound source during rotation, the summation of signals from both TENG units reaches its maximum, inducing a peak EPSC through maximal proton accumulation. Capturing the timing of the current peak enables the real‐time determination of sound direction, establishing bio‐inspired cognitive functions for spatial localization to facilitate continuous environmental monitoring without requiring separate digital computation.

In addition to mimicking the human inner ear, diverse neuromorphic architectures have been explored to implement spatial localization by emulating specialized biological auditory systems. For example, an integrated 2D planar system incorporates an AlN‐based piezoelectric micromachined ultrasonic transducer (pMUT) and a VCM‐based HfO_2_ RRAM to mimic the auditory nervous system of an owl (Figure 8i) [253]. Ultrasonic echoes captured by the pMUT sensor are transformed into single digital voltage spikes (*V*_in_) through an analog front‐end circuit and subsequently applied to an RRAM‐based delay circuit. The architecture adopts an RC network configuration where the RRAM's variable resistance (*R*) and a capacitor (*C*) are connected in series, using the input spike as a driving voltage to charge the capacitor. The fundamental mechanism involves modulating the circuit's time constant (τ  =  *RC*) through the RRAM's analog conductance states, thereby physically controlling the charging slope of the capacitor. A delayed output spike is generated only when the voltage charged at a rate adjusted according to the resistance value of the RRAM reaches the *V*_th_ of the rear inverter. The resistance‐dependent charging delay characteristic enables the high‐fidelity emulation of biological axonal delays, facilitating coincidence detection that transforms temporal disparities between incoming signals into spatial azimuthal information (Figure 8j). By achieving precise acoustic spatial awareness through hardware‐level delay modulation, this architecture serves as a compelling building block for sustainable environmental monitoring systems, such as bioacoustics tracking or detecting external anomalies.

#### 5.2.2 In‐Sensor Computing Systems

**FIGURE 9:**
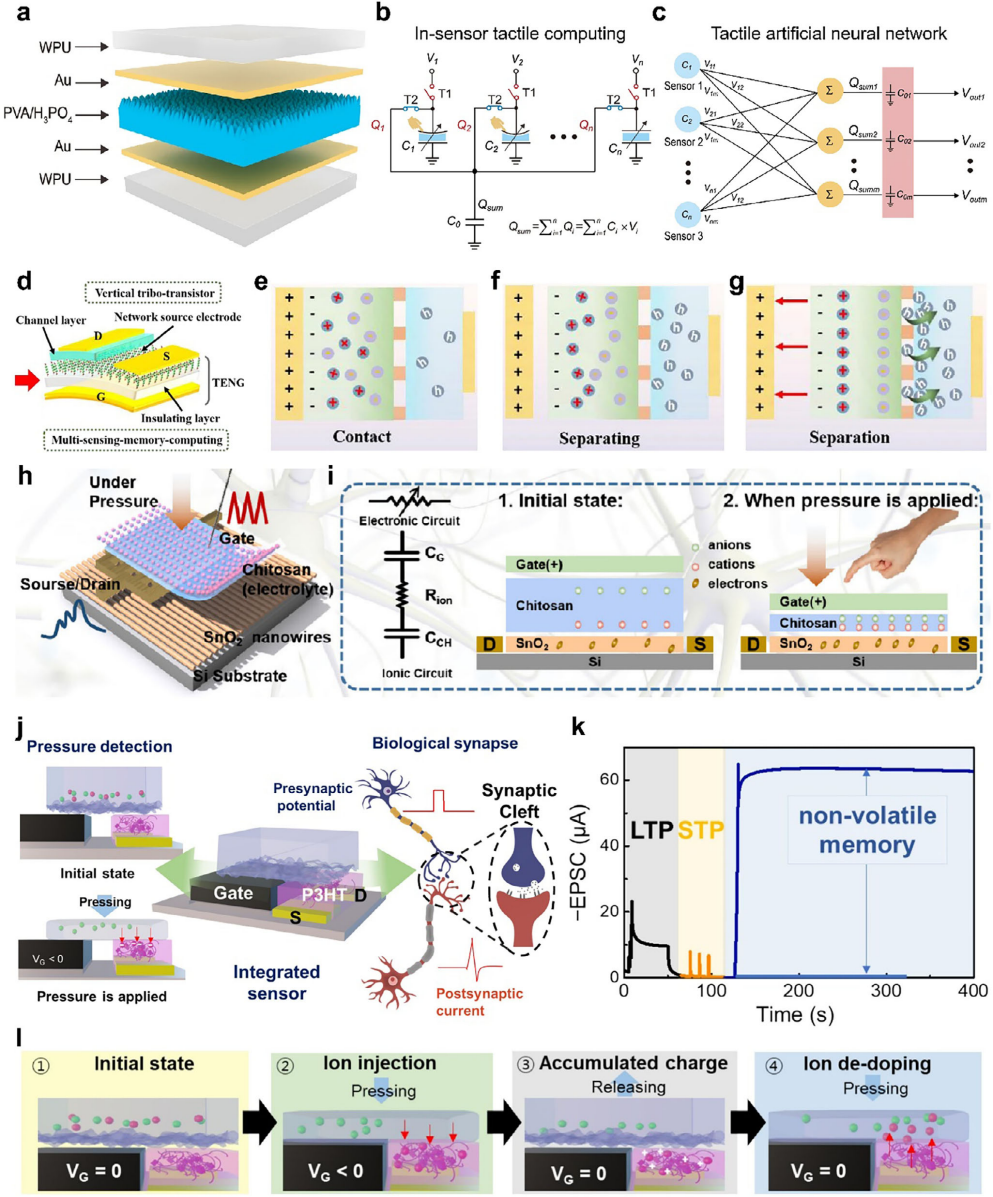
Neuromorphic mechanoreceptors based on in‐sensor integration. (a) Neuromorphic in‐sensor tactile sensing system based on a single‐active‐layer, two‐terminal capacitive pressure sensor and (b) the physical processes of capacitive in‐sensor tactile computing where multiple connected sensors form a subregion to implement in situ multiply‐accumulate (MAC) operations. (c) Architecture of a tactile artificial neural network constructed from these integrated arrays, utilizing an input capacitive pressure sensor vector and a predefined voltage matrix. Reproduced (Adapted) under terms of the CC‐BY license [317]. Copyright 2025, The Authors, published by Springer Nature. (d) Structure of the vertical tribo‐transistor featuring three‐terminal configuration with multiple vertically integrated active layers. (e–g) Charge distributions of source, channel, and drain layers when the gate is (e) in contact with and (f) separated from the IL layer, and (g) charge distribution of each layer at the separation state. Reproduced (Adapted) under terms of the CC‐BY license [335]. Copyright 2022, The Authors, published by Springer Nature. (h) Neuromorphic tactile sensor (NTS) based on a three‐terminal architecture featuring a single active layer. (i) Equivalent circuits and operating principle of the NTS; applied pressure reduces the chitosan film thickness and resistance, enhancing the on‐state efficiency of the field‐effect transistor and consequently modulating the channel current. Reproduced (Adapted) with permission from [336]. Copyright 2025, American Chemical Society. (j) Schematics of artificial tactile perception based on the side‐gated transistor. The left panel shows pressure detection via contact area modulation at the gate/ionogel and ionogel/P3HT interfaces, mimicking the signal transmission of a biological synapse shown in the right panel. (k) Transition characteristics among short‐term plasticity (STP), long‐term plasticity (LTP), and non‐volatile memory within a single multifunctional transistor. (l) Non‐volatile memory mechanism based on ion dynamics. Reproduced (Adapted) with permission from [338]. Copyright 2026, The Authors, published by Elsevier.

While in‐sensor computing has been extensively explored for visual receptors, recent research has increasingly focused on extending the multiply‐accumulate (MAC) computing paradigm to mechanical sensors, such as tactile receptors. However, developing a device that fully functions as both an auditory receptor and a neuron remains challenging, as the triboelectric phenomenon used for detecting sound signals is highly dependent on geometrical structure, which hinders direct computing capabilities. Implementation of in‐sensor MAC computing for tactile stimuli remains in its infancy [248, 317]. Chen et al. demonstrate an in‐sensor tactile computing system utilizing a two‐terminal flexible capacitive pressure sensor array to perform analog signal processing directly at the sensor level, bypassing the need for external digital units [317]. The pressure sensor pixels within the array consist of a waterborne polyurethane (WPU) top substrate, an Au top electrode, a PVA/H_3_PO_4_ sensing layer, an Au bottom electrode, and a WPU bottom substrate (Figure 9a). Upon application of external pressure, contact between the Au electrode and the H_3_PO_4_ sensing layer triggers the migration of free ions to the metal interface, forming a nanometer‐scale EDL. The extremely thin thickness (*d*) of the EDL provides a capacitance per unit area thousands of times higher than conventional capacitors, thereby maximizing device performance. Configuring the unit devices according to the circuit shown in Figure 9b enables analog computation via a two‐stage process of charging and charge sharing. During the charging phase, each sensor pixel (*C*_1_–*C*_n_) accumulates a charge (*Q*_i_ = *C*_i_ *V*_i_) proportional to the product of its stimulus‐induced capacitance and the applied voltage bias (*V*_1_∼*V*_n_). A subsequent switching operation facilitates a charge‐sharing process, aggregating the stored charges at a single common electrode. Consequently, the peak output voltage (*V*_out_) measured across a fixed capacitor (*C*_0_) corresponds to the sum of the products of capacitance and voltage bias ($\left(Q_{sum}=\;\sum C_{i}V_{i}\right)$) for each pixel. This direct physical summation establishes the architecture of a tactile artificial neural network (Figure 9c), where the capacitance array serves as the input vector and the applied voltages represent the predefined weight matrix. The mechanism allows for real‐time analog MAC operations to be physically executed within the sensor network without separate digital conversion. The resulting integrated system exhibits power consumption more than 22 times lower than conventional mixed‐signal sensing platforms. This remarkable energy efficiency paves the way for sustainable green computing, while the inherent physical flexibility of the sensor array highlights its strong potential for reliable operation even in mechanically demanding platforms, such as conformal wearables and soft robotics.

Additionally, research efforts in tactile sensing have expanded toward three‐terminal in‐sensor computing systems to integrate learning and memory functionalities that extend beyond basic MAC operations [335, 336, 337]. For instance, Figure 9d presents a three‐terminal multilayer multisensory‐memory‐computing device that integrates a TENG and a transistor within a vertical organic field effect transistor (VOFET) configuration to realize an advanced artificial perception system [335]. The architecture comprises a detachable Kapton substrate, a detachable Au gate electrode, an ion‐gel dielectric, a MXene‐network‐based source electrode, a PDVT‐10 channel layer, and Au source/drain electrodes. Within this computing device, MXene serves multiple roles as the top electrode of the TENG, the source electrode of the transistor, and a light‐harvesting layer, which collectively enable tactile, auditory, and visual perception. Tactile signal processing in the vertical tribo‐transistor (VTT) relies on the interaction between triboelectric induction potential and ion migration during the contact‐separation cycle between the gate electrode and the ionic liquid. In the initial contact phase, charge accumulates at the interface via electrostatic induction and triboelectrification, yet the system remains in an off state due to a high Schottky barrier (Figure 9e). As the gate electrode begins to separate, the resulting triboelectric potential modulates ion migration within the ion‐gel layer to form an EDL and lower the Schottky barrier height (Figure 9f). Upon complete separation, the dense EDL narrows the barrier width to facilitate hole injection, establishing an on state where current flows vertically from the MXene electrode to the PDVT‐10 channel (Figure 9g). Variations in input signal intensity and frequency drive the transition from STP to LTP, where the synergy of multisensory stimuli allows the firing threshold to be reached more rapidly than with unimodal inputs, effectively recapitulating the signal amplification and integration functions inherent to biological sensory systems.

Du et al. introduce a three‐terminal neuromorphic tactile sensor (NTS) based on a vertically integrated multilayer device by integrating SnO_2_ nanowires with a pressure‐sensitive chitosan ion‐gating layer, successfully fusing pressure sensing and neuromorphic computing within a single architecture (Figure 9h) [336]. As illustrated in Figure 9i, the operating principle relies on the reduction of the chitosan electrolyte layer thickness upon the application of external pressure, which decreases the internal ionic resistance (*R*_ion_) and modifies the potential distribution. The resistance variation directly regulates the efficiency of ion migration and accumulation driven by the gate voltage, thereby enabling the dynamic tuning of EPSC in response to applied pressure level. By substituting pressure stimuli for psychological stress factors, the researchers emulate the biological phenomenon in which cognitive performance improves under high‐stress conditions, proving the efficacy of this bio‐inspired edge computing platform in enhancing learning efficiency and memory retention.

To further advance memory retention capabilities, the incorporation of a micropatterned ionogel into tactile synaptic transistors has been demonstrated as an effective strategy for achieving stable LTP [338]. Figure 9j presents schematics of a side‐gated 3‐terminal transistor integrating a micropatterned ionogel gate dielectric and a poly(3‐hexylthiophene‐2,5‐diyl) (P3HT) semiconductor for artificial tactile perception. The integrated transistor exhibits a broad pressure sensing range from 500 kPa to 8.5 MPa and a tunable sensitivity of up to 58 000 kPa^−1^, while maintaining a high‐conductance state for over 46 000 s without continuous gate bias. In the initial state without external pressure, the channel remains in the OFF state. This occurs because limited interfacial contact at both the gate electrode/ionogel and ionogel/P3HT interfaces results in low capacitance. Upon applying pressure, the elastic ionogel deforms, enhancing the contact across all interfaces. When a gate voltage is concurrently applied, anions in the ionogel migrate into the P3HT channel, transitioning the device to the ON state. Building on this pressure‐switchable ON/OFF behavior, the device dynamically switches between STP, LTP, and non‐volatile memory functions through pressure‐mediated ion dynamics (Figure 9k). The physical deformation of the micropatterned ionogel under appropriate pressures creates structural barriers that prevent ion drift, while the transition between STP and LTP is governed by the applied gate voltage. Under a positive gate voltage, the injected anions rapidly de‐dope from the P3HT channel, resulting in STP, whereas a negative gate voltage sustains anion accumulation within the channel, giving rise to LTP. The non‐volatile memory operation proceeds through four distinct states governed by the interplay between applied pressure and gate voltage (Figure 9l). In the initial state, the absence of external pressure results in minimal contact between the micropatterned ionogel and the P3HT channel, leaving the device in the OFF state. During the programming step, applied pressure expands the ionogel/P3HT contact area, and a simultaneous negative gate voltage drives anion injection into the semiconductor, inducing hole accumulation and switching the device to the ON state. Upon pressure release in the retention state, the reduced interfacial contact area cuts off the ion diffusion pathway back to the ionogel, trapping the injected ions within the channel and sustaining the high‐conductance ON state without any applied power. Finally, the erasing step is achieved by reapplying pressure to restore interfacial contact, followed by an erasing gate voltage that extracts the trapped ions and returns the device to the OFF state. By achieving seamless reconfiguration among transient plasticity and non‐volatile memory states through dual electro‐mechanical modulation, this architecture presents a robust hardware solution for energy‐efficient neuromorphic systems, demonstrating strong potential for applications in human‐machine interfaces, soft robotics, and neuromorphic data processing.

### 5.3 Neuromorphic Chemoreceptors (Olfactory & Gustatory)

#### 5.3.1 Near‐Sensor Computing Systems

**FIGURE 10:**
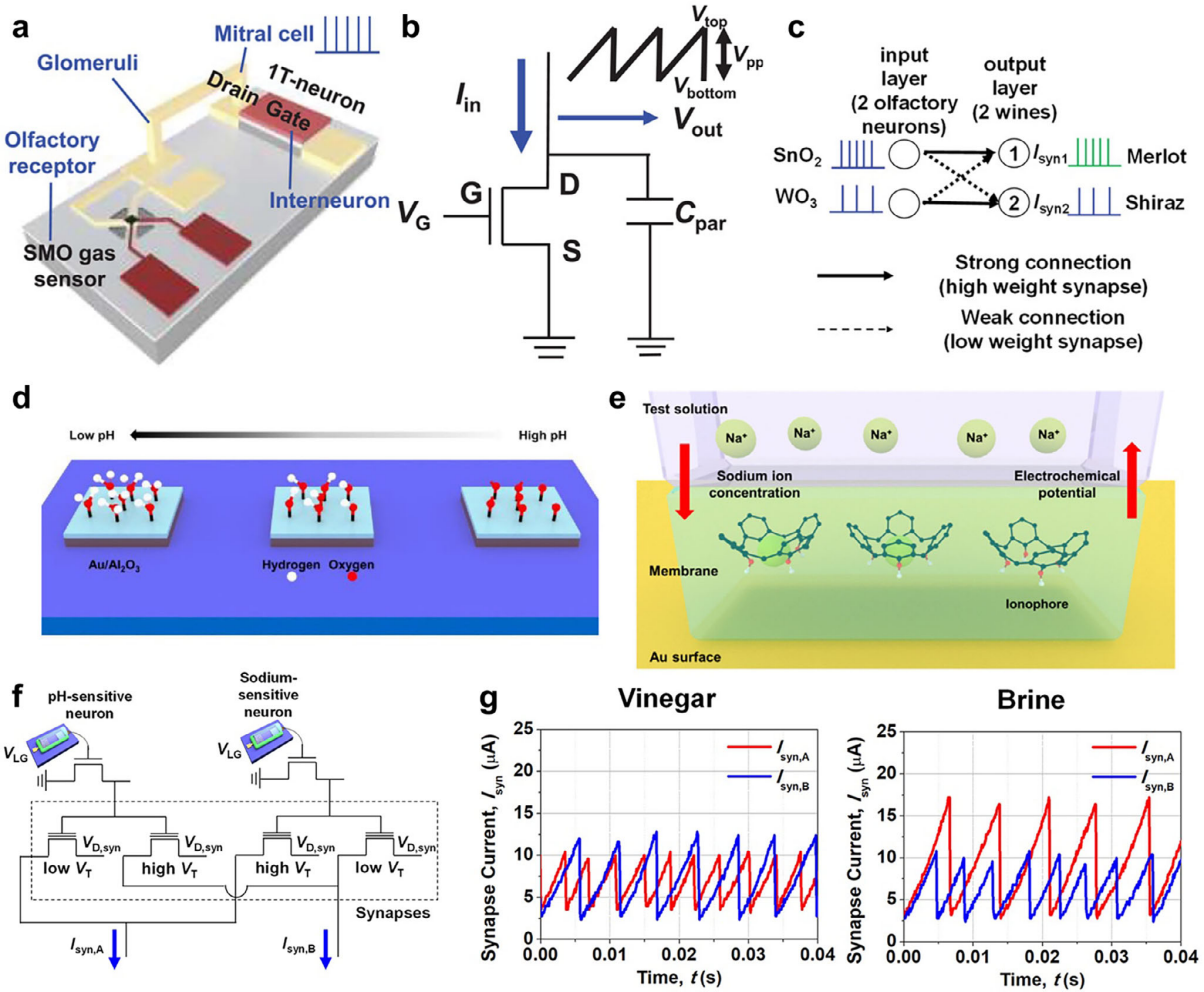
Neuromorphic chemoreceptors based on near‐sensor integration. (a) A proposed artificial olfactory neuron module based on the 2D planar integration of a SMO gas sensor and a MOSFET‐based 1T‐neuron, where the sensor acts as an olfactory receptor. (b) Circuit schematic illustrating the neuron operation; a constant input current (*I*_in_) is applied to the drain electrode, and the output voltage (*V*_out_) is measured at the same node. (c) Architecture of a single‐layer spiking neural network (SNN) utilizing the artificial olfactory neuron modules as input layers for the classification of two distinct wines. Reproduced (Adapted) under terms of the CC‐BY license [340]. Copyright 2022, The Authors, published by John Wiley and Sons. (d, e) Sensing principles of the proposed 2D planar‐integrated gustatory neuromorphic sensor, showing (d) the pH response to hydrogen ions and (e) the sodium response to sodium ions. (f) Circuit diagram of the corresponding E‐tongue hardware, illustrating the transmission of electrical signals from artificial gustatory neurons to homotypic synapses. Synaptic weights are modulated by changing the threshold voltage (*V*_T_) via charge trapping in the Si_3_N_4_ layer (*V*_D,syn_ = 1 V). (g) Measured synaptic currents at output layers A and B (*I*_syn,A_​ and *I*_syn,B_​), where dominant spiking frequencies in layer A or B selectively indicate the detection of vinegar or brine, respectively. Reproduced (Adapted) with permission from [341]. Copyright 2022, American Chemical Society.

Chemical sensors, such as gas and biosensors, for environmental and medical applications require a shift toward miniaturized, low‐power hardware to ensure portability. Despite the significant advancements of neuromorphic architectures in physical sensing domains like vision and touch, near‐sensor computing for olfactory and gustatory systems remains in its early stages [130, 339]. As a foundational step toward addressing this gap, recent efforts have focused on developing hardware that directly transforms chemical stimuli into biologically relevant spike signals. Han et al. develop an artificial olfactory neuron system that integrates a semiconducting metal oxide (SMO) chemoresistive gas sensor with a metal‐oxide‐semiconductor field‐effect transistor (MOSFET) neuron (1T‐neuron) within a single pixel to facilitate simultaneous sensing and spike signal encoding (Figure 10a) [340]. The interaction between target gas molecules and ionosorbed oxygen on the SMO surface modulates the electron‐depleted region, inducing a resistance shift that modulates the *I*_in_, which accumulates in a parasitic capacitor (*C*_par_) to increase the *V*_out_, reproducing the integration characteristics of biological neurons. When V_out_ reaches a specific threshold (*V*_latch_), a rapid firing event occurs via the single‐transistor latch‐up (STL) phenomenon. The frequency‐modulated characteristics exhibit a spike firing frequency (*f*) that is linearly regulated in proportion to the gas concentration (Figure 10b). The architecture replicates essential biological olfactory features such as on/off‐type responses and inhibitory functions while generating unique firing frequencies for different gas types. To demonstrate a practical hardware application, a single‐layer spiking neural network (SNN) is constructed utilizing two distinct artificial olfactory neuron modules (SnO_2_ and WO_3_) as input layers for the classification of two distinct wines (Figure 10c). This architecture implements binary synaptic weights via a 1T1R structure to establish strong and weak connections between the specific sensors and the output layers. Consequently, the integrated hardware successfully classifies the different wines based on their distinct output spiking frequencies, validating the efficacy of the artificial olfactory system.

To detect specific tastes, the authors also develop an artificial gustatory neuron system where the sensing units operate based on distinct ion‐selective mechanisms [341]. The system integrates an ion‐selective electrode (ISE) and a 1T‐neuron at the pixel level to respond to hydrogen ions (sourness) and sodium ions (saltiness). The pH sensing unit operates through the reversible binding of hydrogen ions (H^+^) to the surface, which modifies the surface potential (Figure 10d). Meanwhile, sodium detection involves a sodium‐selective membrane that captures Na^+^ ions, generating a membrane potential change due to the resulting concentration gradient at the membrane interface (Figure 10e). Consistent with the near‐sensor configuration of the electronic nose, the overall circuit is constructed by integrating these distinct units responsible for sourness and saltiness, where ionic information from each unit modulates the *I*_in_ of the 1T‐neuron (Figure 10f). Charges accumulate in the parasitic capacitor until the threshold is reached, at which point firing occurs to encode the data into spike frequencies (*f*). The artificial gustatory neurons classify vinegar and brine by comparing the output spiking frequencies measured at synaptic layers A (*I*_syn,A_) and B (*I*_syn,B_), where a dominant frequency in a specific layer indicates the corresponding liquid (Figure 10g). Simulations utilizing spike data collected from exposing the device to various concentrations of vinegar (pH sensing) and brine (Na^+^ sensing) successfully demonstrate the distinct and clear classification of these substances, proving the potential of the bio‐inspired edge computing platform.

#### 5.3.2 In‐Sensor Computing Systems

**FIGURE 11:**
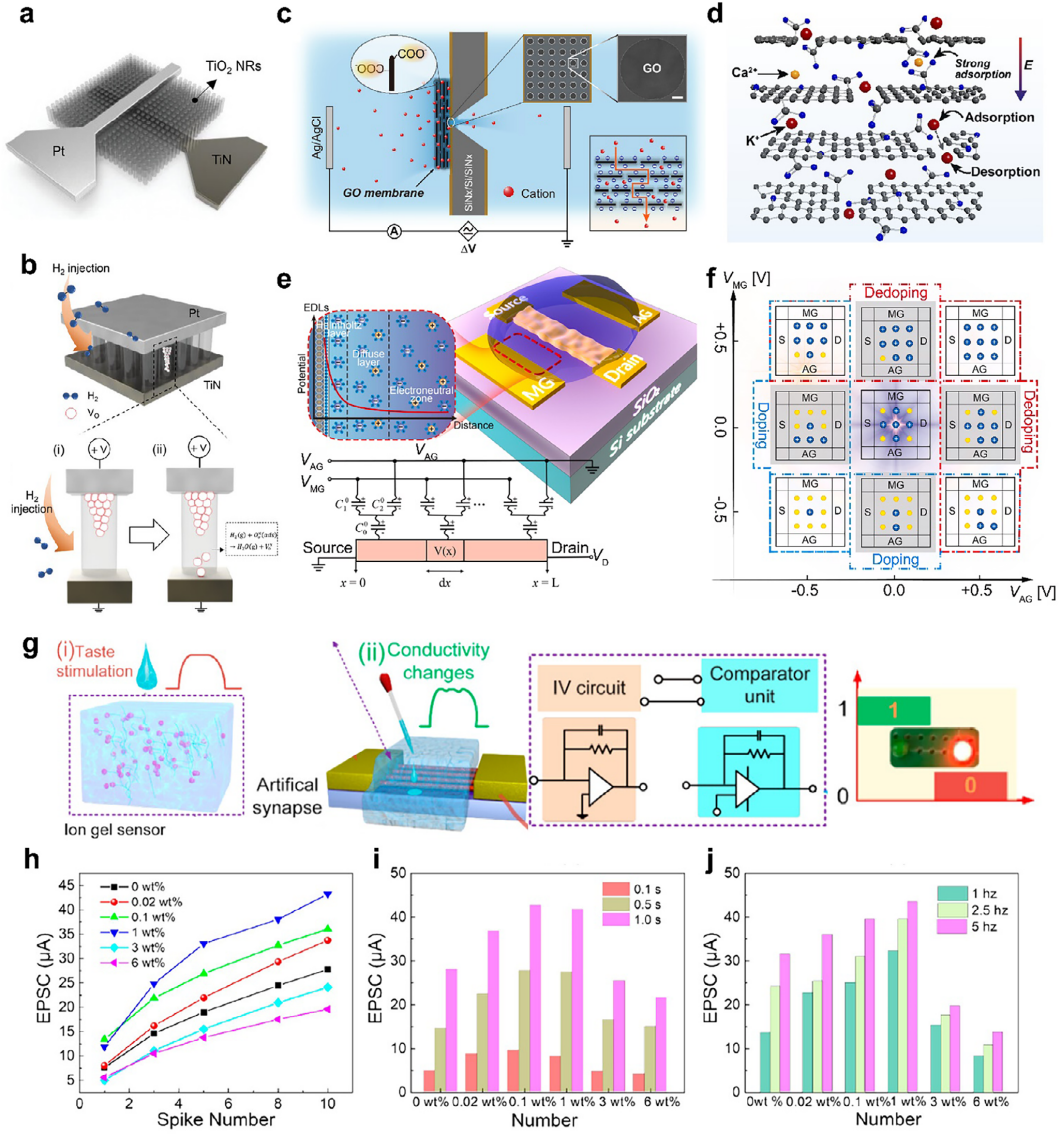
Neuromorphic chemoreceptors based on in‐sensor integration. (a) A two‐terminal chemi‐memristive gas sensor with a crossbar structure (Pt/TiO_2_ NRs/TiN), and (b) the sensor response to H_2_ in (i) the initial state and (ii) after the generation of oxygen vacancies induced by the reaction with H_2_. Reproduced (Adapted) with permission of the Licensor through PLSclear under terms of the CC‐BY‐NC‐ND license [130]. Copyright 2023, The Authors, published by John Wiley and Sons. (c) Schematic illustration of the two‐terminal device configuration featuring a 4 mm × 4 mm GO membrane (SEM scale bar: 500 nm), and (d) the ion dynamics within the GO layers. Reproduced (Adapted) under terms of the CC‐BY‐NC‐ND license [342]. Copyright 2025, The Authors, published by National Academy of Sciences. (e) Dual‐liquid‐gated OECT operating as a three‐terminal gustatory sensor in one‐gate‐only mode. (f) Channel (de)doping states under dual‐liquid‐gating operation. (yellow/blue spheres: holes/cations; S, D, MG, AG: respective electrodes). Reproduced (Adapted) with permission from [343]. Copyright 2021, Elsevier. (g) The artificial gustatory system consists of a chitosan ion‐gel sensor, SnO_2_ NWs artificial synapses, and an execution unit. The ion‐gel sensor perceives different [NaCl] by controlling the inflow numbers of Na^+^, encoding information as spikes that are transported to the brain for processing and generation of outputs to trigger the activities of execution units. (h–j) Dependence of the EPSC in the synaptic device on various stimulus parameters: (h) spike number, (i) spike duration, and (j) spike frequency, measured under different concentrations of salt solution. Reproduced (Adapted) with permission from [344]. Copyright 2023, American Chemical Society.

In‐sensor olfactory systems go beyond the simple SNN signal emulation of existing near‐sensor structures by directly implementing synaptic plasticity, such as STP and LTP, thereby realizing intelligent pre‐processing at the sensor level [130, 342, 343]. For example, Chun et al. propose an artificial olfactory system utilizing chemi‐memristive dynamics based on an oxygen‐vacancy‐mediated ECM mechanism in a two‐terminal Pt/TiO_2_ NR/TiN MIM structure (Figure 11a) [130]. In the HRS, the ruptured conductive filament regions remaining within the TiO_2_ nanorods react with external H_2_ molecules to generate additional oxygen vacancies and release electrons, which directly increases the conductivity of the insulating layer (Figure 11b). When gas injection ceases, the oxygen vacancies are restored by atmospheric oxygen, and the device quickly returns to its initial state (STP). Conversely, repeated stimuli strengthen the filaments, slowing the oxygen recovery rate and leading to a transition into long‐term memory. These oxygen‐vacancy‐mediated dynamics provide fast response speeds at room temperature and hysteresis‐based memory characteristics that distinguish them from those of conventional gas sensors.

Intelligent pre‐processing has also been achieved in in‐sensor gustatory systems. For example, Zhang et al. report a two‐terminal graphene oxide (GO) ion‐sensing memristor device (GO‐ISMD) that integrates sensing and computation by utilizing the ion confinement effect of GO membranes (Figure 11c) [342]. Figure 11d illustrates the interfacial adsorption and desorption processes of ions occurring within the GO nanochannels. As ions repeatedly adsorb to and desorb from functional groups such as hydroxyl and carboxyl groups on the GO surface, the rearrangement of ionic charges is delayed, substantially extending the retention time of the conduction state and exhibiting memristive behavior. By combining the nonlinear response characteristics of the ionic memristors with a reservoir computing architecture, GO‐ISMD successfully identifies basic tastes—such as sweet, salty, sour, and bitter—as well as the complex flavors of liquids like coffee and cola with high accuracy.

In another study, Ji et al. propose a PEDOT:PSS‐based three‐terminal organic electrochemical transistor (OECT) platform that utilizes a dual‐liquid‐gate configuration to freely adjust performance metrics and neuromorphic properties post‐fabrication for gustatory sensing [343]. As depicted in Figure 11e, the system maximizes interfacial capacitance with the electrolyte and effectively modulates channel potential by utilizing two gate electrodes on a plane. Figure 11f shows the initial state of the system with both the main gate (MG) and auxiliary gate (AG) at zero bias (center), a single‐gate mode where only one gate is active (gray cross area), and a dual‐gate mode where both gate potentials combine to determine the electrochemical state of the channel (four corners). The most unique feature of the OECT is the “pre‐de‐doping” effect, where the AG determines the operating range of the MG. Applying a positive voltage to the AG induces de‐doping by pushing cations into the channel through electrostatic repulsion, thereby reducing conductivity. This feature allows for the variable control of threshold voltage (*V*_th_), transconductance (*g*_m_), and the relaxation time of paired‐pulse depression (PPD) by up to 3.7 times, proving the potential for implementing highly plastic artificial synapses.

Moving beyond passive taste recognition, a notable study has demonstrated an artificial gustatory system that effectively merges environmental monitoring with healthcare, incorporating practical health‐monitoring features, such as an excessive‐intake warning function [344]. Figure 11g illustrates the biological gustatory transmission process emulated by an ion‐gating‐based three‐terminal SnO_2_ nanowire synaptic device and an effect‐executive unit, featuring a chitosan ion gel that serves simultaneously as the sensing layer and the gate insulator. As a saline solution permeates the ion gel, the variation in Na^+^ concentration induces variations in ionic conductivity. Such changes modulate the stimulus information delivered to the SnO_2_ NW artificial synapse, causing output spike values to vary in proportion to the ionic concentration. Salt intake can be monitored in real‐time by activating red or green LEDs based on whether the current signal exceeds a threshold. Furthermore, the SnO_2_ nanowire artificial synapse exhibits spatiotemporal plasticity, where the synaptic weight is modulated by various stimulus parameters. The EPSC values and the resulting memory effect are gradually strengthened as the spike number (Figure 11h), spike duration (Figure 11i), and spike frequency (Figure 11j) increase. These plasticity characteristics are significantly influenced by the salt concentration; specifically, while high [NaCl] (>1 wt%) may slow ion migration due to excessive accumulation, the elevated salt levels enhance the storage capacity and memory retention time. Notably, the high surface‐to‐volume ratio of the SnO_2_ nanowires enables ultrasensitive operation, with a minimum responsive voltage as low as ∼1 mV under saline conditions, providing a foundation for low‐power artificial gustatory systems and future green computing platforms.

## 6 Conclusion

In summary, we have provided a comprehensive review of the operating principles and architectures of neuromorphic in‐sensor and near‐sensor computing systems, starting from the drawbacks of conventional sensing architectures. Conventional sensing architectures are often limited by the von Neumann bottleneck—a narrow pipeline connecting physically separated sensing and computing units. This bottleneck causes substantial energy and latency inefficiency by forcing raw or lightly processed sensor outputs to traverse interconnects and data converters before reaching the computing unit [345, 346]. Neuromorphic architectures aim to alleviate this limitation architecturally by producing computation‐ready representations (e.g., events or stateful signals) and embedding synaptic/neuronal functions close to the sensing front‐end [318, 347, 348].

Neuromorphic functionality is ultimately realized by encoding computation into device‐embedded state variables whose evolution and retention mimic synaptic/neuronal behaviors [349]. Across material platforms, external stimuli or electrical programming can drive state evolution through mechanisms such as charge trapping/detrapping, ionic migration and interfacial electrostatics, ferroelectric polarization switching, phase transitions, or filamentary resistive switching [132, 347, 350, 351]. When these mechanisms yield gradual, history‐dependent, and sufficiently retained modulation of conductance or threshold characteristics, the resulting state can directly represent synaptic weight or neuronal excitability, enabling plasticity (e.g., STP/LTP) and event‐like responses without relying on fully digital processing [347, 349].

From the sensing perspective, the modalities covered in this Review can be organized by stimulus and transduction physics into optical (vision), mechanical (touch and hearing), and chemical (olfaction and taste) sensing. Across these sensing front‐ends, each modality relies on distinct receptor and transduction mechanisms to convert external stimuli into measurable electrical or electrochemical signals at the sensor interface (e.g., photocurrent/photovoltage, piezo‐/tribo‐/piezoresistive responses, or adsorption/ion‐mediated modulation) [352]. By establishing these modality‐specific front‐end principles, this Review provides the physical context for sensor–neuromorphic integration: the key design question is how these transduced signals are represented, routed, and processed as the system moves from sensing toward computation, which motivates a structure‐centric taxonomy.

Therefore, we address a persistent challenge: the distinction between near‐sensor and in‐sensor computing is often ambiguous in current research, with device structure, array configuration, and circuit design frequently lumped together under similar labels. To address this, this Review classifies neuromorphic sensor integration strategies primarily by architecture. Near‐sensor approaches are grouped by physical integration structure—the integration scale and interconnect topology that reduce sensor‐to‐compute distance while keeping sensing and computation functionally decoupled—and are divided into board‐level 2D planar integration [249, 250], chip‐level 3D stacking with dense vertical interconnects [260, 262, 263], and pixel‐level circuit integration [265, 268, 269, 270, 271]. Near‐sensor processing offers a practical path to neuromorphic sensing by leveraging mature components and packaging/circuit co‐design. However, because sensing and computation remain functionally separated, conversion and interface costs persist, motivating in‐sensor computing that co‐localizes sensing and state evolution within a single device element [297]. In contrast, in‐sensor approaches are grouped by device integration structure, specifically, terminal configuration and vertical functional coupling, which captures how stimuli directly program device ‐ embedded state variables and how these states are read out within a single sensing element (e.g., two‐terminal direct modulation [278, 279, 280], three‐terminal gating modulation [286, 287, 288], or heterostructure vertical integration with sensing–memory layer coupling [294, 353]). This architecture‐based terminology is adopted because these structural choices most directly determine the dominant bottlenecks and enable consistent cross‐comparison across materials and platforms.

Finally, the bio‐inspired case studies across modalities demonstrate how this architecture‐based classification clarifies what is being integrated and which bottleneck is being targeted for each sensory function. Across various applications, near‐sensor systems provide practical routes to reduce redundant transfer between sensing and computation through system‐level integration, while in‐sensor devices localize sensing and state evolution to directly couple stimulus transduction to synaptic/neuronal state updates. By organizing examples under a consistent structural criterion, this Review enables clearer comparisons of energy–latency gains, conversion overheads, and area–performance trade‐offs across materials and platforms, providing a common language for pinpointing where progress is most needed. More broadly, the field would benefit from the establishment of cross‐modal, standardized benchmarking frameworks that go beyond individual device figures of merit [354, 355]. In addition to reporting device‐level characteristics, future evaluations should incorporate system‐level task performance under application‐relevant scenarios, energy–latency–accuracy trade‐offs assessed at the array or hardware‐system level, and, where possible, open‐source datasets, evaluation protocols, and software/hardware toolchains to enable reproducible and fair comparisons across materials, devices, and architectures [356, 357].

Beyond the modality‐specific bottlenecks discussed above, a critical challenge toward practical neuromorphic sensor systems is system‐level heterogeneous integration. Because sensing layers, memory/synaptic devices, and logic circuits often rely on dissimilar materials and fabrication flows, multi‐material/process compatibility, thermal budget constraints, and interface reliability remain major obstacles [4, 297, 358]. In addition, neuromorphic sensors frequently generate analog, event‐based, or state‐dependent outputs rather than standard digital streams, making mixed‐signal interfacing between sensory front‐ends and downstream computing hardware a persistent challenge [251, 359]. Advanced packaging approaches, including monolithic 3D integration, heterogeneous 3D stacking, and chiplet‐based schemes, therefore become essential not only for physical integration, but also for balancing interconnect density, bandwidth, latency, energy efficiency, and reliability [261, 360, 361].

Going forward, the maturity and bottlenecks of neuromorphic sensors will diverge by modality. Beyond demonstrating fixed plasticity rules, an important frontier for neuromorphic sensor systems is hardware‐enabled adaptive perception and on‐device online learning. For autonomous and context‐aware intelligent agents, sensory hardware must continuously update its internal states in response to changing environments, task demands, and feedback, rather than relying solely on pre‐programmed or offline‐trained weights [362, 363, 364]. Vision has progressed toward system‐level, low‐latency perception using event‐driven front‐ends [365, 366], and the next step is to translate these gains into array‐scale, semiconductor‐compatible platforms with uniform, drift‐resilient state control via defect/trap and interface engineering or ferroelectric/ionic gate stacks [295, 353, 367]. Touch is increasingly evaluated in closed‐loop action (e.g., contact/slip‐driven control), prioritizing mechanically robust, low‐drift transducers and scalable e‐skin arrays that preserve reliable spike/state encoding under repeated deformation [368, 369, 370], enabled by fatigue‐resistant piezo/tribo composites or iontronic elastomers with stable electrochemistry [368, 371, 372]. Auditory neuromorphic sensing is advancing through cochlea‐inspired front‐ends and spiking acoustic neurons [219, 373], yet demanding broader bandwidth and noise robustness through stable mechano‐electrical transduction materials and tighter front‐end/back‐end co‐integration [373, 374, 375]. Chemical modalities face the most severe stability constraints: olfaction must overcome humidity/temperature sensitivity and long‐term drift through selective functional materials and ruggedized packaging [340, 376, 377, 378], while taste additionally demands liquid‐stable, antifouling interfaces where ionic state evolution can be exploited for learning [341, 342, 344].

More fundamentally, progress toward next‐generation neuromorphic sensor systems will require a stronger co‐design mindset that tightly couples computational theory, hardware architecture, and material/device innovation from the outset. Rather than adapting learning algorithms to pre‐existing hardware constraints or retrospectively identifying applications for newly developed devices, future neuromorphic sensor systems should be conceived through a co‐design framework from the outset. Such a framework requires the joint definition of the target sensory task, the desired information representation and learning rule, the circuit‐ and array‐level architecture needed to implement them, and, finally, the material or device state variables required to support stable, low‐noise, and adaptive operation. This requires design strategies in which device physics and architecture are selected according to the form of computation that the system must actually perform, such as event‐driven encoding, temporal integration, local feature extraction, weight updating, or closed‐loop adaptation. In turn, material and device choices should be evaluated not only by isolated figures of merit, but also by whether they meet system ‐ level requirements such as state dynamics, retention characteristics, update linearity, variability tolerance, and integration compatibility at the system level. Such a perspective is particularly important for autonomous sensory agents, where sensing, memory, and decision‐relevant state evolution must be coordinated in real time under changing environmental and task conditions. Cross‐layer co‐design will therefore be essential for translating neuromorphic sensors from isolated device demonstrations into deployable platforms for adaptive, context‐aware, and application‐driven intelligence [15].

## Conflicts of Interest

The authors declare no conflict of interest.

## Data Availability

The data that support the findings of this study are available from the corresponding author upon reasonable request.
